# Low 2012–13 Influenza Vaccine Effectiveness Associated with Mutation in the Egg-Adapted H3N2 Vaccine Strain Not Antigenic Drift in Circulating Viruses

**DOI:** 10.1371/journal.pone.0092153

**Published:** 2014-03-25

**Authors:** Danuta M. Skowronski, Naveed Z. Janjua, Gaston De Serres, Suzana Sabaiduc, Alireza Eshaghi, James A. Dickinson, Kevin Fonseca, Anne-Luise Winter, Jonathan B. Gubbay, Mel Krajden, Martin Petric, Hugues Charest, Nathalie Bastien, Trijntje L. Kwindt, Salaheddin M. Mahmud, Paul Van Caeseele, Yan Li

**Affiliations:** 1 Communicable Disease Prevention and Control Service, British Columbia Centre for Disease Control, Vancouver, British Columbia, Canada; 2 School of Population and Public Health, University of British Columbia, Vancouver, British Columbia, Canada; 3 Clinical Prevention Services, British Columbia Centre for Disease Control, Vancouver, British Columbia, Canada; 4 Department of Biological and Occupational Risks, Institut National de Santé Publique du Québec, Québec (Québec), Canada; 5 Department of Social and Preventive Medicine, Laval University, Québec (Québec), Canada; 6 Department of Molecular Research, Public Health Ontario, Toronto, Ontario, Canada; 7 Family Medicine and Community Health Sciences, University of Calgary, Calgary, Alberta, Canada; 8 Department of Virology, Provincial Laboratory of Public Health, Calgary, Alberta, Canada; 9 Department of Microbiology, Immunology and Infectious Diseases, University of Calgary, Calgary, Alberta, Canada; 10 Communicable Disease Prevention and Control, Public Health Ontario, Toronto, Ontario, Canada; 11 Department of Microbiology, Public Health Ontario, Toronto, Ontario, Canada; 12 Department of Laboratory Medicine and Pathobiology and Department of Paediatrics, University of Toronto, Toronto, Ontario, Canada; 13 Department of Paediatrics, The Hospital for Sick Children, Toronto, Ontario, Canada; 14 Laboratoire de Santé Publique du Québec, Institut National de Santé Publique du Québec, Sainte-Anne-de-Bellevue, Québec, Canada; 15 Département De Microbiologie, Infectiologie et Immunologie, Faculté de médecine, Université de Montréal, Montréal, Québec, Canada; 16 Influenza and Respiratory Virus Section, National Microbiology Laboratory, Winnipeg, Manitoba, Canada; 17 Community Health Sciences and Pharmacy, University of Manitoba, Winnipeg, Manitoba, Canada; 18 Cadham Provincial Laboratory, Manitoba Health, Winnipeg, Manitoba, Canada; 19 Department of Medical Microbiology, University of Manitoba, Winnipeg, Manitoba, Canada; Public Health Agency of Canada, Canada

## Abstract

**Background:**

Influenza vaccine effectiveness (VE) is generally interpreted in the context of vaccine match/mismatch to circulating strains with evolutionary drift in the latter invoked to explain reduced protection. During the 2012–13 season, however, detailed genotypic and phenotypic characterization shows that low VE was instead related to mutations in the egg-adapted H3N2 vaccine strain rather than antigenic drift in circulating viruses.

**Methods/Findings:**

Component-specific VE against medically-attended, PCR-confirmed influenza was estimated in Canada by test-negative case-control design. Influenza A viruses were characterized genotypically by amino acid (AA) sequencing of established haemagglutinin (HA) antigenic sites and phenotypically through haemagglutination inhibition (HI) assay. H3N2 viruses were characterized in relation to the WHO-recommended, cell-passaged vaccine prototype (A/Victoria/361/2011) as well as the egg-adapted strain as per actually used in vaccine production. Among the total of 1501 participants, influenza virus was detected in 652 (43%). Nearly two-thirds of viruses typed/subtyped were A(H3N2) (394/626; 63%); the remainder were A(H1N1)pdm09 (79/626; 13%), B/Yamagata (98/626; 16%) or B/Victoria (54/626; 9%). Suboptimal VE of 50% (95%CI: 33–63%) overall was driven by predominant H3N2 activity for which VE was 41% (95%CI: 17–59%). All H3N2 field isolates were HI-characterized as well-matched to the WHO-recommended A/Victoria/361/2011 prototype whereas all but one were antigenically distinct from the egg-adapted strain as per actually used in vaccine production. The egg-adapted strain was itself antigenically distinct from the WHO-recommended prototype, and bore three AA mutations at antigenic sites B [H156Q, G186V] and D [S219Y]. Conversely, circulating viruses were identical to the WHO-recommended prototype at these positions with other genetic variation that did not affect antigenicity. VE was 59% (95%CI:16–80%) against A(H1N1)pdm09, 67% (95%CI: 30–85%) against B/Yamagata (vaccine-lineage) and 75% (95%CI: 29–91%) against B/Victoria (non-vaccine-lineage) viruses.

**Conclusions:**

These findings underscore the need to monitor vaccine viruses as well as circulating strains to explain vaccine performance. Evolutionary drift in circulating viruses cannot be regulated, but influential mutations introduced as part of egg-based vaccine production may be amenable to improvements.

## Introduction

In Canada, as elsewhere in North America, an early and intense epidemic peak distinguished the 2012–13 influenza season [1]–[5]. Influenza A/H3N2 subtype viruses predominated and were associated with increased outbreak reports from long-term care facilities, exceeding tallies of the prior decade in some regions despite higher immunization coverage among residents and staff in those settings [6], [7]. Consistent with these surveillance observations, mid-season assessment of vaccine performance by the established sentinel monitoring system in Canada showed disappointing vaccine effectiveness (VE) of 45% (95%CI: 13–66%) for the H3N2 component [1], similarly low in the United States [8] and Europe [9]. Although suboptimal vaccine performance has historically been linked to evolutionary drift in circulating viruses, H3N2 viruses in Canada and elsewhere globally were characterized throughout the epidemic as antigenically similar to the prototype virus (A/Victoria/361/2011) recommended as 2012–13 vaccine component by the World Health Organization (WHO) [2]–[5], [10].

To understand low VE despite reports of vaccine match, we conducted further epidemiologic and laboratory investigations in end-of-season analyses. With additional participants and contributing viruses, we estimated VE against circulating strains belonging to both influenza A subtypes and B lineages accompanied by their in-depth genotypic and phenotypic characterization in relation to vaccine components. Specifically, vaccine-virus relatedness was assessed genotypically by determining the amino acid (AA) sequence of established haemagglutinin (HA) antigenic sites and phenotypically through the haemagglutination inhibition (HI) assay. For H3N2, virus characterization was in relation to the A/Victoria/361/2011 prototype strain recommended by the WHO [10], as well as the egg-adapted high growth reassortant strain as per that actually used by manufacturers in vaccine production (hereafter “IVR-165”) [11]. We show that suboptimal VE for the H3N2 component during the 2012–13 season was related to mutations in the egg-adapted IVR-165 vaccine strain, rather than antigenic drift in circulating viruses.

## Methods

### Ethics statement

Associated institutional ethics review boards in each contributing province approve this annual evaluation of influenza VE in Canada based on documented oral consent, including the Behavioural Research Ethics Board of the University of British Columbia, the Conjoint Health Research Ethics Board of the Calgary Health Region of Alberta Health and the University of Calgary, the Health Research Ethics Board of the University of Manitoba, the Health Sciences Research Ethics Board of the University of Toronto and the University Health Network (Ontario) and the Comité d'éthique de santé publique, Ministère de la Santé et des Services sociaux du Québec.

### Epidemiologic

A test-negative case-control design embedded within the routine sentinel surveillance network has been used each year in Canada since 2004 to estimate effectiveness of the annually-reformulated trivalent influenza vaccine (TIV) [1], [12]–[19]. Several hundred practitioners from designated community-based sentinel sites in the five most-populous provinces (British Columbia (BC), Alberta, Manitoba, Ontario and Québec) contribute to annual virologic and VE monitoring. Participating sentinel sites can offer nasal or nasopharyngeal swabs for influenza virus testing to all patients presenting within 7 days of influenza-like illness (ILI) onset. ILI is defined as acute fever and cough illness with one or more of sore throat, arthralgia, myalgia or prostration. Fever is not required for elderly patients aged ≥65 years.

At the time of specimen collection, the attending practitioner also obtains epidemiologic information directly from consenting patients/parents/guardians using a standardized questionnaire affixed to the laboratory requisition. Information includes date of symptom onset, current influenza immunization status and month/year of vaccine receipt, as well as prior TIV (2011–12, 2010–11) and 2009 monovalent A(H1N1)pdm09 vaccine receipt [17]. Details related to special pediatric immunization dosing are not sought. Information on comorbidity is recorded on the questionnaire as ‘yes’, ‘no’ or ‘unknown’ to any one or more of the chronic medical conditions defined by Canada's National Advisory Committee on Immunization as increasing the risk of influenza complications, without specifying the condition [20].

### Immunization

Immunized participants primarily receive vaccine during the regular autumn immunization campaign. Influenza vaccine is provided free of charge to all citizens ≥6 months old in Alberta, Manitoba and Ontario. In BC and Québec vaccine is provided free of charge to high-risk individuals and their close contacts or caregivers [20]; others are also encouraged to receive vaccine but must purchase it. For the 2012–13 season, 70% of the national contractual volume of publicly-funded non-adjuvanted, inactivated TIV that was administered was split virus formulation and the rest was subunit. Live attenuated influenza vaccine was also available for those 2–59 years old, but publicly funded only in the participating provinces of Alberta and Québec. An adjuvanted subunit TIV formulation was also available for the elderly but used only in the participating provinces of BC and Ontario.

For the northern hemisphere's 2012–13 TIV, two of three components were changed from the prior season [10]. The WHO recommended a strain-level change for the H3N2 component to include A/Victoria/361/2011-like prototype virus and a lineage-level change to include B/Wisconsin/1/2010(Yamagata-lineage)-like virus. The A/California/7/2009(H1N1)-like virus (hereafter A(H1N1)pdm09) was retained unchanged since 2009 [10]. Manufacturers substituted the egg-adapted high growth reassortant strains A/Victoria/361/2011(H3N2)-IVR-165, A/California/7/2009(H1N1)-NYMC-X-179A (or X-181) (hereafter “X-179A” or “X-181”) and B/Hubei-Wujiagang/158/2009-NYMC-BX-39 as considered antigenically-equivalent to the WHO-recommended prototype viruses. Of the publicly supplied TIV in Canada, 70% included A/California/7/2009-like antigen derived from X-179A and 30% from X-181.

### Laboratory

Specimens were tested for influenza virus at provincial public health laboratories by real-time reverse-transcription polymerase chain reaction (RT-PCR). All RT-PCR positive specimens were inoculated into mammalian cell culture (Madin Darby canine kidney (MDCK) or rhesus monkey kidney (RMK) (Ontario)) for virus isolation and an aliquot of successfully cultivated virus, generally after single passage, was submitted to the National Microbiology Laboratory (Canada's influenza virus reference laboratory) for characterization by haemagglutination inhibition (HI) assay [21]. Currently, an 8-fold or greater reduction in post-infection ferret HI-antibody titre raised to a given reference strain and tested against a field isolate constitutes meaningful antigenic distinction between reference and test viruses, although previously a threshold of 4-fold or greater titre reduction had been applied [21].

For H3N2 viruses, HI characterization was undertaken not only relative to the A/Victoria/361/2011 virus passaged in MDCK cells with whole HA identical to the WHO-recommended MDCK-passaged vaccine prototype, but also relative to the egg-passaged version with whole HA identical to the IVR-165 reassortant vaccine strain. The former was conducted using turkey erythrocytes and validated with guinea pig erythrocytes; the latter was conducted with guinea pig erythrocytes directly [21].

A subset of sentinel H3N2 HA1 and A(H1N1)pdm09 HA1/HA2 genes from viruses detected across the season and contributing to VE analysis were sequenced for phylogenetic and pair-wise AA identity comparison according to methods described in **Text S1**. Virus was sequenced from culture isolates (Ontario, per above) or original patient specimens (all provinces including Ontario in the event virus could not be cultivated). Genotypic findings were interpreted in relation to corresponding phenotypic findings based on HI antigenic characterization. For this analysis we referred to established antigenic site maps which for H3 consist of 131 AA residues across antigenic sites A–E as enumerated in **Table S1** [18], [19], [22] and for H1 consist of 50 AA residues across antigenic sites Sa, Sb, Ca1, Ca2, and Cb as also enumerated in **Table S1** [19], [23].

Influenza B viruses were characterized at the lineage- and/or strain-level by HI, phylogenetic analysis or an influenza B-lineage-specific one-step conventional RT-PCR assay [24]. Because antigenic site maps for influenza B have not yet been established, further gene sequencing and pair-wise identity analysis were not undertaken for influenza B.

### VE analysis

A specimen collected between November 1, 2012 (week 44) and April 30, 2013 (week 18) was considered a case if it tested positive for influenza virus and a control if it tested negative for all influenza types/subtypes. Patients for whom the timing of vaccination was unknown or <2 weeks before symptom onset, or for whom comorbidity was unknown were excluded. We estimated the odds ratio (OR) for medically-attended, laboratory-confirmed influenza in vaccinated versus non-vaccinated participants by logistic regression with adjustment for clinically-relevant confounders. Per previous VE analyses from this sentinel system, covariates included age, comorbidity, province, week of specimen collection and the interval between ILI onset and specimen collection [1], [12]–[19]. VE was calculated as [1-_adjusted_OR]×100. We also separately assessed VE in patients without comorbidity, by age category and by prior immunization history.

## Results

### Participant profile

**Figure 1:**
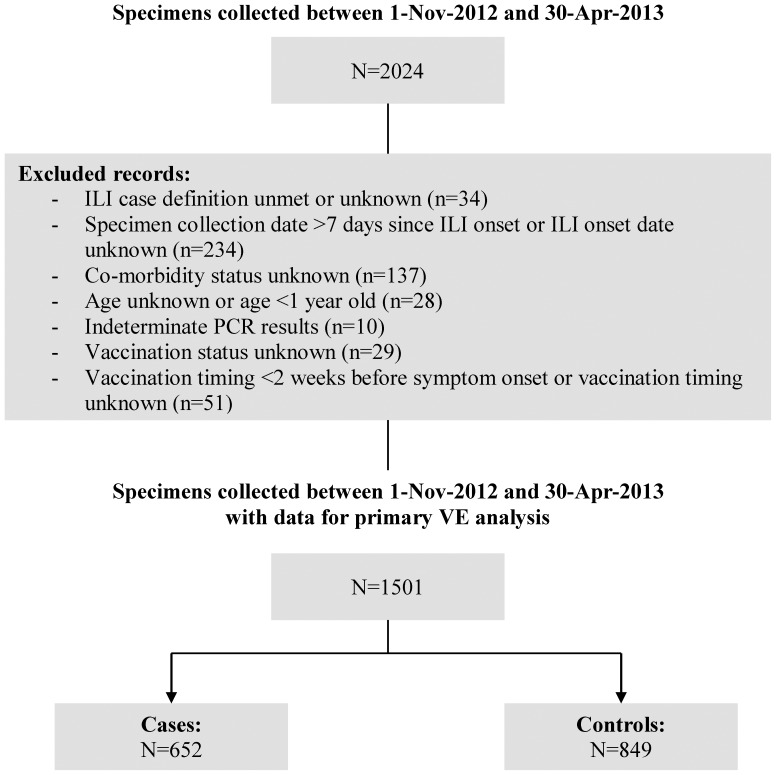
Specimen exclusion for influenza vaccine effectiveness analysis, Canada, 2012–13 sentinel surveillance system. NOTE: exclusions shown here in stepwise fashion to arrive at total case and control tally (i.e. those meeting multiple exclusion criteria are counted on the basis of the first exclusion criterion met in the list shown). Missing collection dates were imputed as the laboratory accession date minus two days, the average time period between collection date and laboratory accession date for records with valid data for both fields.

There were 1501 participants included in final 2012–13 VE analysis (**Figure 1**). Similar to previous participation in our sentinel network, adults 20–49 years of age comprised the greatest proportion (680/1501; 45%) (**Table 1**) [1], [14]–[19]. Overall, 16% (107/664) of cases and 30% (263/888) of controls reported receipt of 2012–13 TIV (p<0.01). After applying exclusion criteria related to immunisation timing, 15% of cases and 26% of controls were considered immunized (p<0.01) (**Table 1**). Only a minority of participants reported receipt of live vaccine overall or among children, or adjuvanted formulation for the elderly (**Table 1**). The proportion of controls immunised is comparable to that of previous VE analyses [1], [14]–[16], [18], [19] and to population immunization coverage separately reported by the Canadian Community Health Survey (CCHS) (∼30%) [25]. The proportion with comorbidity (22%) was also comparable to previous seasons and to CCHS estimates (∼15–20%) (**Table 1**) [1], [14]–[19], [26].

**Table 1:** Profile of participants included in primary influenza VE analysis, 2012–13, Canada.

Characteristics		Case (test-positive)	Control (test-negative)	Total
		N = 652; n (%)	N = 849; n (%)	N = 1501; n (%)
**Age group (years)**	**1–8**	104 (16)	96 (11)	200 (13)
	**9–19**	98 (15)	86 (10)	184 (12)
	**20–49**	279 (43)	401 (47)	680 (45)
	**50–64**	118 (18)	177 (21)	295 (20)
	**≥65**	53 (8)	89 (10)	142 (9)
	**Median age in years (range)**	33 (1–92)	37 (1–95)	35 (1–95)
**Female sex**		369 (57)	505 (59)	874 (58)
**Comorbidity** a		112 (17)	187 (22)	299 (20)
**Received 2012–13 TIV** b	**≥2 weeks before symptom onset**	95 (15)	224 (26)	319 (21)
Among:	**those without comorbidity**	55 (10)	138 (21)	193 (16)
	**those with comorbidity**	40 (36)	86 (46)	126 (42)
Among:	**1–8 years**	5 (5)	18 (19)	23 (12)
	**9–19 years**	0 (0)	10 (12)	10 (5)
	**20–49 years**	36 (13)	73 (18)	109 (16)
	**50–64 years**	26 (22)	57 (32)	83 (28)
	**≥65 years**	28 (53)	66 (74)	94 (66)
Adjuvanted vaccine (≥65 years old)	**Yes**	11 (39)	19 (29)	30 (32)
	**No**	4 (14)	26 (39)	30 (32)
	**Unknown**	13 (46)	21 (32)	34 (36)
**Received prior influenza vaccine**	**2011–12 TIV** c	148/619 (24)	262/784 (33)	410/1403 (29)
	**2010–11 TIV** d	151/596 (25)	267/752 (36)	418/1348 (31)
	**2009 A(H1N1)pdm09 vaccine** e **^,^** f	240/556 (43)	331/709 (47)	571/1265 (45)
**Specimen collection interval (days)**	**≤4**	522 (80)	623 (73)	1145 (76)
	**5–7**	130 (20)	226 (27)	356 (24)
	**Median interval in days (range)**	3 (0–7)	3 (0–7)	3 (0–7)

TIV = trivalent influenza vaccine; VE = vaccine effectiveness.

a . Including any one or more of heart, pulmonary, renal, metabolic, blood, cancer, or conditions that compromise immunity or the management of respiratory secretions, or morbid obesity [20].

b . For the 2012–13 season, of 319 participants reporting vaccine receipt ≥2 weeks before symptom onset, 298 reported this was given through injection, 5 through nasal spray (all children except one) with route of administration unspecified for 16.

c . Children <2 years of age in 2012–13 were excluded from 2011–12 vaccine uptake analysis as they may not have been vaccine-eligible during the fall 2011–12 immunization campaign on the basis of age <6 months.

d . Children <3 years of age in 2012–13 were excluded from 2010–11 vaccine uptake analyses.

e . In Canada, AS03-adjuvanted monovalent A(H1N1)pdm09 vaccine comprised >95% of doses distributed [17].

f . Children <4 years of age in 2012–13 were excluded from monovalent A(H1N1)pdm09 vaccine uptake analyses.

The majority of those considered immunized in 2012–13 also reported prior immunization: 83/91 (91%) cases and 180/206 (87%) controls were immunized in 2011–12 (p = 0.34); 74/85 (87%) cases and 162/199 (81%) controls were immunized in both 2011–12 and 2010–11 (p = 0.24); and 67/83 (81%) cases and 149/189 (79%) controls received the 2009 monovalent A(H1N1)pdm09 vaccine (p = 0.72).

### Influenza detection

**Figure 2:**
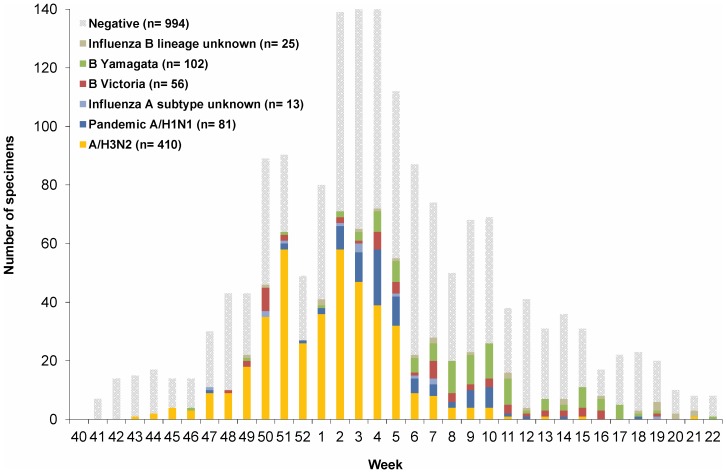
Influenza specimens by week and subtype, 2012–13 sentinel surveillance period (N = 1682). NOTE: excludes specimens from patients failing to meet the influenza-like illness case definition or unknown; specimens collected >7 days after influenza-like illness onset or interval unknown; comorbidity unknown; age unknown or <1 year and influenza test results unavailable or inconclusive on typing. Missing collection dates were imputed as the laboratory accession date minus two days, the average time period between collection date and laboratory accession date for records with valid data for both fields. One specimen diagnosed with both A/H3N2 and A(H1N1)pdm09 in week 2 is not presented in the graph. Vaccine effectiveness analysis spans week 44 to week 18.

The 2012–13 season showed an early November rise and December/January peak in H3N2 activity followed by greater A(H1N1)pdm09 and influenza B contributions thereafter (**Figure 2**). Overall, influenza virus was detected in 652/1501 (43%) specimens tested (**Table 2**). For the 626/652 (95%) influenza detections for which influenza A/subtype and influenza B/lineage could be determined, 394 (63%) were H3N2, 79 (13%) were A(H1N1)pdm09, and one was a dual H3N2 and A(H1N1)pdm09 co-infection; 98 (16%) belonged to the B/Yamagata vaccine-lineage and 54 (9%) belonged to the B/Victoria non-vaccine-lineage (**Table 2**). The proportion immunized by age and influenza type, subtype and lineage is shown in detail in **Table S2**.

**Table 2:** Laboratory profile, 2012–13 sentinel season.

		Alberta	British Columbia	Manitoba	Ontario	Québec	Total
Specimens included		n (%)	n (%)	n (%)	n (%)	n (%)	n (%)
**Influenza tested (N)**		**450**	**319**	**114**	**337**	**281**	**1501**
Influenza negative		267 (59)	185 (58)	77 (68)	193 (57)	127 (45)	849 (57)
Influenza positive	**All influenza positive**	183 (41)	134 (42)	37 (33)	144 (43)	154 (55)	652 (43)
	**A positive**	122 (67)	102 (76)	28 (76)	114 (79)	119 (77)	485 (74)
	**B positive**	61 (33)	32 (24)	9 (24)	30 (21)	35 (23)	167 (26)
Influenza A positive	**A/H3N2**	95 (78)	84 (82)	24 (86)	87 (76)	104 (87)	394 (81)
	**A(H1N1)pdm09**	21 (17)	17 (17)	2 (7)	25 (22)	14 (12)	79 (16)
	**A/H3N2 & A(H1N1)pdm09**	0	0	1 (4)	0	0	1 (1)
	**Subtype unknown**	6 (5)	1 (1)	1 (4)	2 (2)	1 (1)	11 (2)
Influenza B positive	**B/Yamagata (vaccine)**	27 (44)	15 (47)	1 (11)	26 (87)	29 (83)	98 (59)
	**B/Victoria (non-vaccine)**	27 (44)	14 (44)	7 (78)	3 (10)	3 (9)	54 (32)
	**Lineage unknown**	7(12)	3(9)	1(11)	1(3)	3(9)	15(9)
**HI Characterization (post-infection ferret anti-sera raised against reference virus tested against field isolate)**							
**H3N2 Reference Virus**	**A/Victoria/361/2011 (MDCK)** a	2	40	0	17	73	132
	**<4-fold reduced titre**	2 (100)	38 (95)	0	11 (65)	37 (51)	88 (67)
	**≥4-fold reduced titre**	0	2 (5)	0	6 (35)	36 (49)	44 (33)
	**≥8-fold reduced titre**	0	0	0	0	0	0
	**A/Victoria/361/2011 (egg)** b	0	0	0	0	1	1
	**<4-fold reduced titre**	0	0	0	0	0	0
	**≥4-fold reduced titre**	2 (100)	40 (100)	0	16 (100)	72 (100)	130
	**≥8-fold reduced titre**	2 (100)	40 (100)	0	16 (100)	71 (99)	129 (99)^c^
**A(H1N1)pdm09 Reference Virus**	**A/California/7/2009-like**	1	8	0	24	7	40
**Influenza B Reference Virus**	**B/Wisconsin/01/2010 (Yamagata)** d	22 (48)	11 (52)	0	15 (83)	25 (89)	73 (65)
	**B/Brisbane/60/2008 (Victoria)** e	24 (52)	10 (48)	0	3 (17)	3 (11)	40 (35)

TIV: trivalent influenza vaccine; HI: haemagglutination inhibition assay.

a . H3N2 prototype reference strain recommended as 2012–13 TIV component by the World Health Organization (WHO), as passaged in Madin Darby canine kidney cells; assessed using turkey erythrocytes, validated with guinea pig erythrocytes.

b . 2012–13 H3N2 vaccine strain as passaged in eggs and with HA1 sequence identical to the A/Victoria/361/2011 IVR-165 egg-adapted high growth reassortant vaccine strain; assessed based on guinea pig erythrocytes.

c . Nineteen of the 129 viruses (19%) manifesting ≥8-fold reduction had been collected from vaccinated participants, comparable to the proportion immunized among H3 detections overall (17%) and among whom 12/19 (63%) showed 16-fold and 4/19 (21%) showed 32-fold reduction.

d . 2012–13 TIV component.

e . 2011–12 TIV component.

### VE estimates

Crude and adjusted-VE estimates are provided in **Table 3**. Overall VE was 50% (95%CI: 33–63%) and against influenza A was 45% (95%CI: 24–60%). Both estimates were driven by the predominant H3N2 activity during the 2012–13 season for which VE was 41% (95%CI: 17–59%). VE was 59% (95%CI: 16–80%) against A(H1N1)pdm09. Against influenza B, VE was higher at 68% (95%CI: 44–82%): 67% (95%CI: 30–85%) for B/Yamagata vaccine-lineage and 75% (95%CI: 29–91%) for B/Victoria non-vaccine lineage viruses.

**Table 3:** Primary and restricted analysis - influenza vaccine effectiveness based on sentinel system in Canada 2012–13 season.

	Vaccine Effectiveness % (95% Confidence Interval)						
Covariates and adjustment	Any Influenza	Influenza A and Subtype specific			Influenza B and Lineage specific		
		Any Influenza A	A/H3N2	A/H1N1pdm09	Any Influenza B	B/Yamagata (vaccine)	B/Victoria (non-vaccine)
**Primary analysis N total**	1501	1334	1244	929	1016	947	903
**[n Cases; n vaccinated]**	[652; 95]	[485; 78]	[395; 66]	[80; 10]	[167; 17]	[98; 9]	[54; 5]
**(n Controls; n vaccinated)**	(849; 224)	(849; 224)	(849; 224)	(849; 224)	(849; 224)	(849; 224)	(849; 224)
Unadjusted	52 (38–64)	47 (29–60)	44 (24–59)	60 (21–80)	68 (47–81)	72 (43–86)	72 (28–89)
Age (1–8, 9–19, 20–49, 50–64, ≥65 years)	51 (35–63)	46 (26–60)	44 (22–60)	56 (10–79)	68 (44–82)	67 (31–85)	76 (32–92)
Comorbidity (yes/no)	51 (35–63)	45 (27–59)	43 (22–58)	59 (19–80)	66 (43–80)	71 (40–86)	68 (18–88)
Province (BC, AB, MB, ON, QC)	52 (37–63)	46 (28–60)	43 (23–58)	59 (20–80)	69 (47–81)	72 (43–86)	72 (28–89)
Specimen collection interval (≤4 d/5–7 d)	52 (37–63)	46 (28–60)	42 (21–58)	62 (25–81)	68 (46–81)	72 (43–86)	71 (25–89)
Week of illness onset	52 (37–63)	45 (27–59)	41 (20–57)	62 (24–81)	69 (48–82)	73 (45–87)	71 (27–89)
Age, comorbidity, province, interval, week	50 (33–63)	45 (24–60)	41 (17–59)	59 (16–80)	68 (44–82)	67 (30–85)	75 (29–91)
**Restricted to participants with no comorbidity**							
**N total; n Cases; n Controls**	1202; 540; 662	1059; 397; 662	984; 322; 662	728; 66; 662	805; 143; 662	745; 83; 662	710; 48; 662
Adjusted^a^	60 (43–72)	59 (38–72)	53 (28–69)	80 (40–93)	70 (40–85)	69 (25–88)	68 (−4–90)
**Restricted to participants age 1–19 years old**							
**N total; n Cases; n Controls**	384; 202; 182	315; 133; 182	301; 119; 182	193; 11; 182	251; 69; 182	228; 46; 182	203; 21; 182
Adjusted^b^	87 (65–95)	84 (53–95)	87 (55–96)	NE	91 (35–99)	88 (7–98)	NE
**Restricted to participants age 20–49 years old**							
**N total; n Cases; n Controls**	680; 279; 401	622; 221; 401	567; 166; 401	451; 50; 401	459; 58; 401	429; 28; 401	424; 23; 401
Adjusted^c^	31 (−8–56)	32 (−10–58)	17 (−40–51)	56 (−17–84)	32 (−60–71)	10 (−181–71)	54 (−103–90)
**Restricted to participants age ≥50 years old**							
**N total; n Cases; n Controls**	437; 171; 266	397; 131; 266	376; 110; 266	285; 19; 266	306; 40; 266	290; 24; 266	276; 10; 266
Adjusted^d^	47 (17–66)	35 (−6–60)	32 (−15–59)	52 (−51–85)	65 (22–84)	73 (11–92)	79 (4–96)

BC = British Columbia, AB = Alberta, MB = Manitoba, ON = Ontario, QC = Québec; d = days; NE = not estimable owing to sparse data.

a . Adjusted for age (1–8, 9–19, 20–49, ≥50 years), province, interval, week.

b . Adjusted for age (1–8, 9–19 years), comorbidity, province, interval, week; except B/Yamagata not adjusted for province.

c . Adjusted for comorbidity, province, interval, week; except B/Victoria not adjusted for province.

d . Adjusted for age (50–64, ≥65 years), province, interval, week; except A(H1N1)pdm09, influenza B, B/Victoria, B/Yamagata not adjusted for province.

VE estimates were generally increased with restriction to those without comorbidity and among children, but reduced with restriction to adults only, notably those 20–49 years of age (**Table 3**). Repeat immunization had varying effects: those who had received both 2012–13 and 2011–12 TIV had lower VE estimates against H3N2 than those who received 2012–13 TIV alone (**Table S3**). Conversely, those immunized both seasons showed higher protection against both influenza B/lineages. In each of these sub-analyses, however, confidence intervals were broad and overlapping. We particularly lacked statistical power related to the A(H1N1)pdm09 component, although in separately-grouped indicator analysis there was suggestion of comparable or higher 2012–13 TIV protection in those with prior receipt of the same unchanged A(H1N1)pdm09 vaccine antigen, including the 2009 monovalent formulation (**Table S4**).

### Influenza genetic and antigenic characterization

To assess the impact of genotypic and phenotypic differences on VE estimates, we compared antigenic site sequence analysis and HI characterization of MDCK cell- and egg-passaged influenza A vaccine and circulating viruses.

#### A(H3N2)

Of 395 H3N2 infections diagnosed by PCR, 152 (38%) viruses contributed to genotypic analysis with specimen collection dates spanning November 10 to April 10 including 56 (37%) with November–December, 92 (60%) with January–February and 4 (3%) with March–April collection. The vast majority of these viruses (143/152; 94%) belonged to the same phylogenetic clade 3C as did both the MDCK-passaged, WHO-recommended A/Victoria/361/2011 prototype virus and the egg-adapted IVR-165 reassortant strain actually used in vaccine production (**Figure S1**).

However, more detailed sequence analysis revealed three antigenic site AA differences between the IVR-165 and the WHO-recommended A/Victoria/361/2011 prototype. These three mutations in IVR-165, located close to the receptor binding site, include H156Q and G186V substitutions at antigenic site B, and S219Y mutation at antigenic site D. Conversely, the HA1 gene of all 152 circulating viruses, like their 2011–12 vaccine and circulating predecessors [19], shared AA identity with the MDCK-cell-passaged prototype at these three positions (**Table 4**). In association with the IVR-165 antigenic-site mutations, we observed 16-fold reduction in HI antibody titre raised against the egg-passaged version when tested against the MDCK-cell-passaged prototype. This is consistent with the 32-fold reduction also reported by the WHO in its comparison between IVR-165 and the WHO-recommended prototype [10]. Also similar to the WHO report [10], there was no reduction for antibody raised to the MDCK-cell-passaged virus when tested in reverse against the egg-passaged version in two-way HI comparison.

**Table 4:** Haemagglutinin antigenic site differences in circulating H3N2 viruses relative to the 2012–13 egg-adapted A/Victoria/361/2011 IVR-165^a^ high growth reassortant vaccine strain.

H3N2 Hemagglutinin																																	
Vaccine Reference Virus = Victoria 361 IVR-165																																	
Antigenic Site		C				E				D		A	B	A			B						D			E	C				Clade	# of AA differences^b^	% AA identity^b^
HA1 Position		45	48	53	54	62	67	88	94	103	121	124	128	140	142	145	156	157	186	192	193	198	219	226	230	262	278	280	304	312			
A/Victoria/210/2009 (X-187)^c^		N	**T**	D	S	**K**	I	V	Y	P	N	S	T	I	R	N	**H**	L	V	I	F	S	**S**	I	I	S	N	E	A	N	1	11	91.6%
A/Victoria/361/2011 (MDCK)		N	I	D	S	E	I	V	Y	P	N	S	T	I	R	N	**H**	L	**G**	I	F	S	**S**	I	I	S	N	E	A	S	3C	3	97.7%
**A/Victoria/361/2011 (IVR-165)** a		N	I	D	S	E	I	V	Y	P	N	S	T	I	R	N	Q	L	V	I	F	S	Y	I	I	S	N	E	A	S	3C	-	-
A/Texas/50/2012 (MDCK)^d^		N	I	D	S	E	I	V	Y	P	N	S	**N**	I	R	N	**H**	L	**G**	I	F	**P**	**S**	I	I	S	**K**	E	A	S	3C	6	95.4%
A/Texas/50/2012 (X-223)^d^		N	I	D	S	E	I	V	Y	P	N	S	**N**	I	R	N	**H**	L	V	I	F	**P**	**F**	**N**	I	S	**K**	E	A	S	3C	6	95.4%
**British Columbia**	**N**																																
A/British Columbia/020/2012	10												**A**		**G**	**S**	**H**		**G**				**S**				**K**				3C	7	94.7%
A/British Columbia/021/2012	12												**A**		**G**	**S**	**H**		**G**				**S**				**K**				3C	7	94.7%
A/British Columbia/023/2012	1																**H**	**S**	**G**				**S**				**K**				3C	5	96.2%
A/British Columbia/002/2013	6															**S**	**H**		**G**				**S**				**K**				3C	5	96.2%
A/British Columbia/022/2013	1												**A**		**G**	**S**	**H**		**G**				**S**				**K**	**K**			3C	8	93.9%
A/British Columbia/023/2013	1														**S**	**S**	**H**		**G**				**S**				**K**				3C	6	95.4%
A/British Columbia/025/2013	1									**Q**						**S**	**H**		**G**				**S**				**K**				3C	6	95.4%
**Alberta**	**N**																																
A/Alberta/053/2012	2																**H**		**G**				**S**				**K**				3C	4	96.9%
A/Alberta/054/2012	1	**S**	**T**	**N**					**H**								**H**		**G**			**A**	**S**		**V**			**A**	**D**	**N**	6	12	90.8%
A/Alberta/056/2012	21						**V**									**S**	**H**		**G**				**S**				**K**				3C	6	95.4%
A/Alberta/059/2012	13															**S**	**H**		**G**				**S**				**K**				3C	5	96.2%
A/Alberta/060/2012	4				**G**											**S**	**H**		**G**				**S**				**K**				3C	6	95.4%
A/Alberta/02/2013	1										**S**					**S**	**H**		**G**				**S**				**K**				3C	6	95.4%
A/Alberta/03/2013	2						**V**									**S**	**H**		**G**				**S**			**N**	**K**				3C	7	94.7%
A/Alberta/06/2013	1												**A**		**G**	**S**	**H**		**G**				**S**				**K**				3C	7	94.7%
A/Alberta/24/2013	1						**V**									**S**	**H**		**G**		**S**		**S**				**K**				3C	7	94.7%
**Manitoba**	**N**																																
A/Manitoba/001/2012	2						**V**									**S**	**H**		**G**				**S**				**K**				3C	6	95.4%
A/Manitoba/003/2012	5															**S**	**H**		**G**				**S**				**K**				3C	5	96.2%
A/Manitoba/004/2012	3												**A**		**G**	**S**	**H**		**G**				**S**				**K**				3C	7	94.7%
A/Manitoba/01/2013	1															**S**	**H**		**G**				**S**								3C	4	96.9%
**Ontario**	**N**																																
A/Ontario/030/2012	9															**S**	**H**		**G**				**S**				**K**				3C	5	96.2%
A/Ontario/031/2012	2											**R**	**A**		**G**	**S**	**H**		**G**				**S**				**K**				3C	8	93.9%
A/Ontario/001/2013	1										**S**					**S**	**H**		**G**				**S**				**K**				3C	6	95.4%
A/Ontario/004/2013	1	**S**	**T**	**N**					**H**								**H**		**G**			**A**	**S**		**V**			**A**		**N**	6	11	91.6%
A/Ontario/005/2013	8												**A**		**G**	**S**	**H**		**G**				**S**				**K**				3C	7	94.7%
A/Ontario/015/2013	1															**S**	**H**		**G**			**A**	**S**				**K**				3C	6	95.4%
A/Ontario/018/2013	1														**K**	**S**	**H**		**G**				**S**				**K**				3C	6	95.4%
A/Ontario/021/2013	1																**H**		**G**				**S**				**K**				3C	4	96.9%
A/Ontario/038/2013	1												**S**		**G**	**S**	**H**		**G**				**S**				**K**				3C	7	94.7%
**Quebec**	**N**																																
A/Quebec/011/2012	22												**A**		**G**	**S**	**H**		**G**				**S**				**K**				3C	7	94.7%
A/Quebec/012/2012	1							**I**								**S**	**H**		**G**				**S**				**K**				3C	6	95.4%
A/Quebec/016/2012	4	**S**	**T**	**N**					**H**								**H**		**G**			**T**	**S**		**V**			**A**		**N**	6	11	91.6%
A/Quebec/019/2012	3																**H**	**S**	**G**				**S**				**K**				3C	5	96.2%
A/Quebec/020/2012	2	**S**	**T**	**N**					**H**								**H**		**G**			**A**	**S**		**V**			**A**		**N**	6	11	91.6%
A/Quebec/021/2012	2															**S**	**H**		**G**				**S**				**K**				3C	5	96.2%
A/Quebec/034/2012	1	**S**	**T**	**N**					**Q**								**H**		**G**			**T**	**S**		**V**			**A**		**N**	6	11	91.6%
A/Quebec/038/2012	2												**A**		**G**	**S**	**H**		**G**	**V**			**S**				**K**				3C	8	93.9%
A/Quebec/14/2013	1													**M**		**S**	**H**		**G**				**S**				**K**				3C	6	95.4%
A/Quebec/26/2013	1					**G**							**A**		**G**	**S**	**H**		**G**				**S**				**K**				3C	8	93.9%

N = number of sentinel viruses with that sequence. Bold font signifies amino acid (AA) substitutions compared with IVR-165. Clade designation, number of antigenic site differences and percent antigenic site pairwise identity are also displayed. Only the 31/131 antigenic site positions showing differences between circulating H3N2 viruses and IVR-165 are displayed. AA sequences at those positions for other recent vaccine viruses are also displayed.

a . IVR-165 is the egg-adapted high growth reassortant strain substituted by manufacturers for the MDCK-passaged A/Victoria/361/2011 (H3N2) prototype virus recommended as 2012–13 vaccine component by the World Health Organization (WHO) designated here as A/Victoria/361/2011 (MDCK).

b . Number of antigenic site AA differences and percent antigenic site identity relative to IVR-165. Percent identity derived as per **Text S1**.

c . A/Victoria/210/2009 (X-187) is the egg-adapted high growth reassortant strain used by manufacturers for the 2011–12 influenza vaccine for the northern hemisphere., shown for historic comparison.

d . A/Texas/50/2012 (MDCK) and (X-223) are the WHO-recommended prototype and egg-adapted high growth reassortant strains, respectively, for the 2013–14 influenza vaccine for the northern hemisphere, shown for added comparison.

There were 132 H3N2 isolates successfully cultured for HI characterization, with collection dates spanning November 20 to April 10, including 61 (46%) with November–December, 68 (52%) with January–February and 3 (2%) with March–April collection. None of these isolates showed ≥8-fold reduction in antibody titre relative to the MDCK-cell-passaged strain, indicating that circulating viruses spanning the H3N2 season were antigenically-equivalent to the WHO-recommended prototype (**Table 2**). Conversely, all but one H3N2 isolate showed ≥8-fold reduction relative to the egg-passaged strain, including viruses collected from season start and with more than half of the circulating viruses (72/130) showing 16-fold and one-quarter (34/130) showing 32-fold titre reduction. This indicates that circulating viruses spanning the season were antigenically distinct from IVR-165.

**Figure 3:**
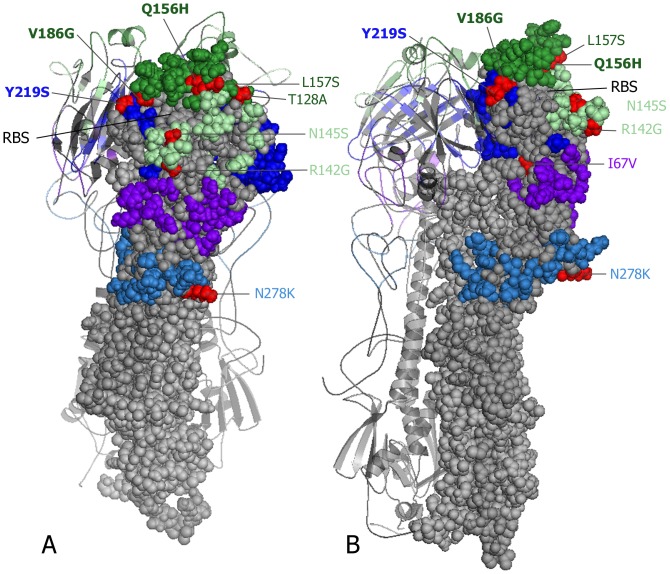
Three-dimensional model of antigenic-site differences between circulating H3N2 viruses and the 2012–13 egg-adapted A/Victoria/361/2011 IVR-165 high growth reassortant vaccine strain. One HA1 monomer is shown with five previously defined antigenic site residues of A–E colored in light green, dark green, light blue, dark blue and purple, respectively, mapped onto a related crystal structure (A/X-31(H3N2), PDB, 1HGG) [27] using PyMOL [28]. The most prevalent antigenic site amino acid differences between circulating clade 3C viruses in Canada relative to the egg-adapted A/Victoria/361/2011 IVR-165 vaccine reassortant strain are shown in red and labelled with coloured font representing their antigenic sites, viewed from the front (A) or side (B). Three amino acid differences (Q156H, V186G and Y219S) are owing to mutation in the egg-adapted IVR-165 vaccine strain rather than circulating viruses which instead share identity with the MDCK-passaged WHO reference prototype at these positions. RBS indicates approximate location of the receptor-binding site.

This antigenic separation between IVR-165 and circulating viruses was primarily associated with mutations in the egg-adapted vaccine. The most prevalent antigenic-site differences between circulating viruses and IVR-165 are illustrated in **Figure 3** [27], [28], including the 3 differences from vaccine at positions 156, 186 and 219 resulting from IVR-165 mutation but not evident in relation to the WHO-recommended prototype. The majority of the circulating clade 3C viruses (134/144; 93%) showed a total of 5-7AA antigenic-site differences relative to IVR-165 (95–96% vaccine identity) (**Table 4**). Fewer showed 4AA (4/144;3%) or 8AA (6/144;4%) total differences relative to IVR-165. Nine other circulating H3N2 viruses belonged to clade 6 and showed 11–12AA antigenic-site differences from IVR-165 (91–92% vaccine identity). However, among the 73 H3N2 viruses spanning November 20 to April 10 for which both genotypic (sequencing) and phenotypic (HI) characterization were undertaken there was a similar distribution of up to 32-fold-reduction in HI titres relative to the egg-passaged strain. This was true regardless of the nature or number of additional AA mutations in circulating clade 3 or clade 6 viruses beyond the three vaccine mutations (**Table 5**).

**Table 5:** Distribution of fold-reduction in haemagglutination inhibition (HI) titres relative to the 2012–13 egg-passaged H3N2 strain by nature and location of additional amino acid (AA) mutations present in HA1 antigenic sites of circulating viruses.

		Specific HA1 antigenic site AA mutations in circulating viruses by fold-reduction in HI titre relative to the egg-passaged H3N2 vaccine strain^a^			
Number of additional antigenic site AA mutations in circulating viruses^b^ (N = number of viruses)	Clade	4-fold	8-fold	16-fold	32-fold
		n/N (%)	n/N (%)	n/N (%)	n/N (%)
**1 (N = 1)**	3C	—	—	—	1/1 (100%)
					**N278K [C]**
**2 (N = 16)**	3C	—	3/16 (19%)	8/16 (50%)	5/16 (31%)
			N278K +	N278K +	N278K +
			**N145S [A]** (×3)	**N145S** (×4) or	**N145S** (×5)
				**L157S [B]** (×4)	
**3 (N = 4)**	3C	—	—	1/4 (25%)	3/4 (75%)
				N278K +	N278K +
				N145S +	N145S +
				**V88I** **[E]** (×1)	**S54G [C]** (×1) or
					**S198A [B]** (×1) or
					**I140M [A]** (×1)
**4 (N = 44)**	3C	1/44 (2%)	10/44 (23%)	25/44 (57%)	8/44 (18%)
		N278K +	N278K +	N278K +	N278K +
		N145S +	N145S +	N145S +	N145S +
		**R142G [A]** +	**R142G** +	**R142G** +	**R142G** +
		**T128A [B]**	**T128A** (×9) or	**T128A** (×25)	**T128A** (×8)
			**T128S** (×1)		
**5 (N = 2)**	3C	—	—	1/2 (50%)	1/2 (50%)
				N278K +	N278K +
				N145S +	N145S +
				R142G +	R142G +
				T128A +	T128A +
				**E62G [E]** (×1)	**I192V [B]** (×1)
**8 (N = 6)**	6	—	—	3/6 (50%)	3/6 (50%)
				**N45S [C]** +	**N45S** +
				**I48T [C]** +	**I48T** +
				**D53N [C]** +	**D53N** +
				**I230V [D]** +	**I230V** +
				**E280A [C]** +	**E280A** +
				**S312N [C]** +	**S312N** +
				**Y94H/Q [E]** +	**Y94H/Q** +
				**S198A/T [B]** (×3)	**S198A/T** (×3)

HA1 = haemagglutinin 1 protein.

Mutations are highlighted in bold in the first row that they are represented in the table.

HA1 antigenic site positions [A–E] affected are annotated in bold the first time they appear.

“(x n)” following a specified amino acid residue indicates the number of viruses with that specific mutation.

a . The 2012–13 egg-passaged H3N2 strain used in haemagglutination inhibition (HI) assay was identical in its HA1 to the egg-adapted A/Victoria/361/2011-IVR-165 high growth reassortant vaccine strain.

b . In addition to the 3 AA differences (at positions 156, 186, and 219) present in the egg-passaged H3N2 strain used in the HI assay and the egg-adapted A/Victoria/361/2011 IVR-165 high growth reassortant vaccine strain.

Taken together, these findings suggest that H3N2 viruses were antigenically equivalent to the WHO-recommended prototype but antigenically distinct from the IVR-165 vaccine component and that vaccine mismatch was predominantly related to mutations in the egg-adapted vaccine strain, rather than evolutionary drift in circulating viruses.

#### A(H1N1)pdm09

Sequence analysis showed that the egg-adapted X-179A (and X-181) vaccine reassortant strain bore no antigenic-site AA mutations relative to the WHO-recommended prototype, and of the 40 A(H1N1)pdm09 isolates spanning November 19 to March 22 characterized by HI (85% collected in January–February), all were antigenically-similar to the vaccine strain (**Table 2**). Fifty-seven circulating A(H1N1)pdm09 viruses spanning November 19 to March 8 (85% collected in January–February) were also sequenced, of which 55 (96%) belonged to clade 6 (**Figure S2**). In September 2013, the European Centre for Disease Prevention and Control (ECDC) further divided clade 6 viruses into three genetic subgroups such that 1/55 (2%), 2/55 (4%) and 52/55 (95%) of our sentinel clade 6 A(H1N1)pdm09 viruses during 2012–13 belong to clade 6A, 6B and 6C, respectively [29]. The majority of the sentinel clade 6 viruses (47/55; 85%) showed 3AA antigenic-site substitutions relative to X-179A/X-181 and 94% vaccine identity with fewer showing 2AA (7/55) or 4AA (1/55) substitutions and two clustered within clade 7 with 3-4AA mutations (**Table S5**).

The genetic profile of circulating A(H1N1)pdm09 viruses differs from 2011–12 when 90% of sequenced viruses clustered within clade 7, bearing the same 2AA mutations shared by all subsequent 2012–13 clade 6/7 viruses (S185T/P and S203T) [19] but with greater additional genetic diversity observed in 2012–13. Other antigenic site mutations in 2012–13, located close to the receptor-binding site, include 21/57 (37%) viruses with R205K (seen in clade 5 sequences in 2011–12), 17/57 (30%) with A141T, and 8/57 (14%) with A186T (**Table S5,** **Figure S3**) [30].

## Discussion

The sentinel surveillance system in Canada directly links genotypic and phenotypic characterization of circulating influenza viruses to epidemiologic measurement of VE in order to better understand vaccine protection in the context of vaccine-virus relatedness. For the 2012–13 season, we used this platform to investigate protection provided by the H3N2 vaccine component, for which VE was first reported to be suboptimal (45%) in mid-season publication [1] despite widespread laboratory reporting that circulating viruses remained antigenically well conserved [2]–[5], [10]. In end-of-season analysis we corroborate mid-season epidemiologic findings of low VE (41%) and reconcile these with laboratory findings. Through detailed gene sequencing and HI comparison we show that reduced vaccine protection during the 2012–13 season was related to mutations in the egg-adapted H3N2 high growth reassortant strain used in vaccine production, not antigenic drift in circulating viruses.

Vaccine match/mismatch to explain variable VE has historically focused on diversity and drift in circulating viruses and their evolving antigenic distance from the corresponding vaccine component. Here, we broaden that perspective to include the potentially serious implications of even a few AA mutations introduced through egg-adaptation of the WHO-recommended cell-passaged prototype virus. The early provision of an egg-adapted high growth reassortant version of the WHO-recommended prototype is a fundamental requirement of influenza vaccine manufacturing, needed for further high-yield growth in embryonated hens' eggs as part of annual mass production [31]. However, in a variety of animal models, mammalian cell-derived H1 and H3 viruses have been shown to induce more cross-reactive antibody response and better protection than corresponding egg-adapted variants bearing as few as 1–2AA mutations [32]–[35]. Such changes with egg passage, particularly if located near the HA receptor-binding site, have been shown to dramatically alter vaccine antigenicity, immunogenicity and efficacy [32]–[35]. Located closest to the receptor-binding site, mutations at antigenic sites A, B and D of the H3 globular head are typically considered most consequential [36] and the immuno-dominance of antigenic site B is particularly emphasized among more recent H3N2 strains [37].

In that regard, we highlight mutations present in the 2012–13 egg-adapted high growth reassortant IVR-165 vaccine strain relative to the WHO-recommended H3N2 prototype at positions 156 and 186 of site B, and at position 219 of site D. These mutations were associated with altered vaccine antigenicity and low VE even while antigenic integrity of circulating viruses was maintained. Site B positions 156 and 186 are well-known egg-adaptation sites [38], [39] but QH (i.e. glutamine-histidine) variation at position 156 has additionally been highlighted as one of two HA residues (in addition to position 155) responsible for the significant A/Fujian/411/02 (H3N2) antigenic drift and the suboptimal VE reported during that dramatic 2003–04 influenza epidemic [38]. In more recent publication, substitutions at just seven of the 131 H3N2 A–E antigenic site residues, located exclusively in antigenic sites A (position 145) and B (positions 155, 156, 158, 159, 189 and 193) have been highlighted as responsible for all major H3N2 antigenic cluster transitions since 1968 [40]. Of these, only position 156 distinguishes circulating viruses in 2012–13 from IVR-165 and not from the WHO-recommended cell-passaged prototype. Although our circulating viruses also manifest substitution at position 145 in relation to both IVR-165 and the WHO prototype (Table 4), this difference did not exacerbate fold-reduction in HI titres in relation to the former, and did not alter antigenic equivalence in relation to the latter.

Divergence at position 156 due to vaccine mutation may have therefore been particularly influential in reducing antibody recognition and neutralization of circulating viruses, compromising VE. Of note, the A/Texas/50/2012 egg-adapted high growth reassortant strain (X-223) selected as replacement for the 2013–14 TIV also manifests substitutions at positions 186 and 219 (Table 4) but no longer at position 156. With that 156 homology, X-223 shows antigenic equivalence (≤4-fold reduction in HI titres) relative to the MDCK cell-passaged A/Victoria/361/2011 strain that is once again the WHO-recommended prototype for the 2013–14 vaccine [10]. It is concerning, however, that 74% (90/122) of our sentinel viruses collected during the 2012–13 season still showed ≥8-fold reduction in HI titres when further tested with anti-sera raised against the egg-passaged A/Texas/50/2012 strain and 24% (29/122) showed ≥16-fold reduction. X-223 manifests additional antigenic site B (T128N, S198P) and D (I226N) mutations relative to A/Victoria/361/2011 and IVR-165, different also from our circulating viruses. Ongoing monitoring of H3N2 vaccine-virus relatedness and impact on VE thus remain critical.

These findings related to mutation in egg-adapted vaccine strains highlight a need for in-depth monitoring not only of circulating viruses but also of annual vaccine constituents. In reporting vaccine match/mismatch, both real time [2]–[5] and in retrospective reviews [41], the comparator vaccine referent (whether the original MDCK or egg-passaged WHO prototype, egg-adapted high growth reassortant strain or further egg-propagated virus) should be specified. This would enable more accurate understanding of the correlation between antigenic match and VE. Until now, commentaries on VE as it relates to vaccine match have focused on the similarity between circulating virus and the WHO recommended reference—an approach that our study shows can lead to incorrect conclusions about similarity to the actual vaccine component used and the anticipated vaccine protection on that basis. While evolutionary drift in circulating viruses cannot be regulated, mutations that are introduced as part of egg-based vaccine production may be amenable to improvements. To determine the antigenic relationship between two viruses proposed as equivalent vaccine candidates, ferret anti-sera to both viruses (e.g. the egg- and MDCK-passaged) must be used in a “two-way” HI test [21]. In the current study, and in follow-up report by the WHO [10], two-way HI testing revealed ≥8-fold reduction in antibody titre raised to the egg-passaged strain when tested against the MDCK-passaged version, but this titre reduction was not observed when tested in reverse (anti-sera raised to the MDCK-passaged strain tested against the egg-passaged virus). One-way HI testing consisting only of the latter more cross-reactive direction does not show the antigenic difference between IVR-165 and the recommended cell-passaged A/Victoria/361/2011 prototype [11]. Routine display of two-way HI testing for candidate vaccine viruses could reveal this issue in advance of vaccine production and use, and enable public health programs to more broadly respond to its potential implications.

Specific virus-host interactions are also relevant to consider in interpreting VE findings. In sub-analyses, VE was higher for all TIV components in young participants <20 years of age and those without comorbidity, but for H3N2 was further reduced in adults and those with history of prior immunization. Random variation associated with small sample size in subgroup analysis has to be the first consideration in explaining these differences. Beyond that, hypotheses to explain variability of repeat vaccine effects include varying positive or negative interference from pre-existing antibody determined by antigenic distance across successive vaccine and circulating variants as well as differential neutralization efficiency of affected HA epitopes [36], [42]. Ongoing monitoring of genetic variability across vaccines and circulating viruses may improve resolution and refine our measure of antigenic distance relevant to the effects of repeat immunization. The previous season's 2011–12 TIV included the antigenically-distinct H3N2 predecessor strain A/Victoria/210/2009-X-187 bearing 11AA differences from IVR-165 and 91.6% cross-vaccine identity (Table 4). We lacked statistical power to explore the influence of prior immunization stratified by age or comorbidity but among immunized controls, a comparable proportion <20 years versus 20–49 years of age were immunized the prior year (15/18, 83% versus 55/68, 81%; p = 0.19), greater among those with than without comorbidity (79/82, 96% versus 101/124, 82%; p<0.01). However, single cross-season differences in prior immunization do not necessarily reflect the cumulative lifetime effects of vaccine- or virus-induced antibody that may also be influential. Such immunologic interactions are important to explore but most studies, including our own, lack the required power to assess their intricate effects.

Our end-of-season analyses provide other noteworthy insights. Relative to the WHO-recommended prototype, there were no antigenic-site mutations in the 2012–13 egg-adapted A(H1N1)pdm09 X-179A (or X-181) vaccine strain. Circulating viruses were shown by HI to remain antigenically similar to A/California/07/2009, retained as vaccine antigen since 2009. Nevertheless, our point estimate of VE in 2012–13 (59%; 95%CI: 16–80%) was reduced compared to the prior 2011–12 season (80%; 95%CI: 54–92%) [19]. Confidence intervals around each of these estimates are broad and overlapping such that conclusions regarding VE trends across seasons cannot be drawn. However, the genetic profile of circulating A(H1N1)pdm09 viruses in 2012–13 was more diverse than 2011–12, particularly in relation to the receptor binding site. Ongoing monitoring of differences in the contributing mix of genetic variants across seasons and their correlation with variation in VE may be relevant given recent resurgence of A(H1N1)pdm09 activity [43] and retention of the same vaccine antigen for the northern hemisphere's 2013–14 TIV. After including the same B/Victoria-lineage as TIV component across three consecutive seasons (2009–10 to 2011–12), the WHO recommended a lineage-level switch to B/Yamagata-containing vaccine for the 2012–13 TIV. We found comparable VE estimates of about 70% for co-circulating Yamagata- and Victoria-lineages this season. Immunologic recognition across influenza B/lineages might be anticipated given the greater AA similarity across the HA1 of influenza B/lineages (∼90% pairwise identity) than across influenza A H1/H3 subtypes (∼35% pairwise identity) [44]. We have previously demonstrated cross-lineage immunologic interactions and differential vaccine effects based on prior original priming and subsequent boost exposure histories [44]–[46]. Population heterogeneity in B/lineage exposures with differential recall of immunologic memory (i.e. complex cohort effects) may be evident in cross-lineage protection with varying age-related and prior immunization effects (**Table S3**). Recent meta-analysis has summarized cross-lineage TIV effectiveness from eight randomized controlled trials, mostly among adults, at 52% (95%CI: 19–72%) [47]. Precise quantification and better understanding of the variability in cross-lineage VE for influenza B will be crucial in assessing the incremental cost-benefit of proposed quadrivalent vaccine formulations to replace TIV.

There are limitations to this study. We routinely assess vaccine-relatedness through gene sequencing and HI characterization of contributing viruses from across the season, but this represents only a proportion of all influenza virus detections. Systematic differences in viruses available for characterization or sequencing cannot be ruled out—an issue for all laboratory-based surveillance. We did not directly access IVR-165 but instead, MDCK- and egg-passaged viruses used in HI assays were derived from reference strains provided by the United States Centers for Disease Control and Prevention, confirmed through sequence analysis to be identical in their HA to the A/Victoria/361/2011 WHO prototype and to the IVR-165 reassortant strains, respectively. Two-way HI comparison of these viruses was consistent with WHO report [10]. Working-age adults and repeat vaccine recipients typically comprise the majority of our sample; virologic and VE findings may not be generalizable to other populations. In Canada, universal health care coverage addresses barriers to access that may exist in other countries. We include only participants meeting a specified ILI definition presenting within 7 days of ILI onset helping to standardize for health-care seeking behaviour and illness severity. However, patient and clinician discretion is still incorporated into the decision to test. Because no national immunization registry documenting influenza vaccine receipt exists in Canada, self-report of vaccine status cannot be further validated but has been shown elsewhere to be reliable [48] and was comparable here to separate survey estimates of coverage for seasonal TIV (∼30%) [25] and 2009 monovalent pandemic H1N1 (∼40%) [49] vaccine. We do not collect information on manufacturer's brand of vaccine administered, but most of the seasonal vaccine publicly funded in Canada is non-adjuvanted inactivated split virion product; other formulations are available such as live attenuated vaccine preferentially recommended for children or adjuvanted subunit vaccine approved for the elderly [20], but as shown in **Table 1**, these products contributed little to our overall or age-stratified 2012–13 VE analyses. Although we conducted subset analyses of VE, the reduced sample size and wide confidence intervals in sub-analyses preclude definitive conclusions. Validity of VE estimates derived by the test-negative approach has been demonstrated previously through modelling [50] and more recently empirically through direct comparison to gold-standard per-protocol analysis of the same randomized-controlled trial datasets [51]. Our participant profiles are comparable to previous estimates from the sentinel system and community surveys in Canada. Although we observed no obvious flags for concern, as with any observational design, we cannot rule out residual bias and confounding.

In summary, our findings underscore the need to monitor vaccine viruses as well as circulating strains to explain vaccine performance. Evolutionary drift in circulating viruses cannot be regulated, but virus changes introduced as part of egg-based vaccine production may be amenable to improvements. In that regard a better understanding of specific mutations related to egg-adaptation and most influential upon vaccine protection is needed. We highlight the immuno-epidemiologic complexity that may further influence VE, including agent-host interactions and prior antigenic exposures. This complexity is daunting to consider but critical to confront in improving influenza prevention and control. Finally, we show that sentinel surveillance structures can efficiently and reliably link detailed virologic and epidemiologic observations at the molecular, individual and population levels in support of programmatic and scientific insights and should be considered a core requirement for ongoing influenza vaccine monitoring and evaluation.

## Supporting Information

Figure S1 **Phylogenetic tree of influenza A/H3N2 viruses, sentinel system 2012–13.** A maximum-likelihood phylogeny of the 152 sentinel viruses in the context of globally isolated 2012–2013 H3N2 viruses and recent vaccine components (n = 93) based on nucleotide alignment of the haemagglutinin HA1 domain is shown. Vaccine components and previously reported clades are labelled; sentinel viruses are coloured by province of origin.
(PDF)

Figure S2 **Phylogenetic tree of influenza A(H1N1)pdm09 viruses, sentinel system 2012–2013.** A maximum-likelihood phylogeny of the 57 sentinel viruses in the context of globally isolated 2012–2013 A(H1N1)pdm09 viruses and recent vaccine components (n = 77) based on nucleotide alignment of the haemagglutinin HA1/HA2 domains is shown. Vaccine components and previously reported clades are labelled; sentinel viruses are coloured by province of origin. In September 2013, the European Centre for Disease Prevention and Control (ECDC) further divided clade 6 viruses into three genetic subgroups such that 1/55 (2%), 2/55 (4%) and 52/55 (95%) of the sentinel clade 6 A(H1N1)pdm09 viruses displayed belong to clade 6A, 6B and 6C. Subclade details are displayed in **Table S5** [29].
(PDF)

Figure S3 **Three-dimensional model of antigenic-site mutations in circulating A(H1N1)pdm09 viruses relative to the 2012–13 egg-adapted A/California/04/2009 X-179A high growth reassortant vaccine strain.** Three-dimensional structures of the trimeric haemagglutinin (HA) protein were constructed using the crystal structure of the A/California/04/2009(H1N1) HA (PDB, 3LZG) [30] Amino acid residues of the Sa, Sb, Ca1, Ca2 and Cb antigenic regions on the molecular surface (A: front view; B: top view) are colour-coded purple, green, yellow, pink and blue respectively and amino acid substitutions in circulating viruses relative to X-179A are labelled with coloured text representing their antigenic site positions. The three most prevalent mutations found in this study are coloured red (R205K site Ca1, A141T site Ca2, and A186T site Sb). Clade characteristic mutations S185T (representative for S185T/P) in antigenic site Sb and S203T (not visible in the figure) in antigenic site Ca are coloured cyan. RBS indicates approximate location of the receptor-binding site.
(TIFF)

Table S1 **Influenza A H3 and H1 antigenic site maps.**
(PDF)

Table S2 **Proportion of participants who received 2012–13 TIV by age and influenza type, subtype and lineage.**
(PDF)

Table S3 **Prior 2011–12 trivalent influenza vaccine (TIV) effects on current 2012–13 TIV effectiveness.**
(PDF)

Table S4 **Prior 2011–12 trivalent influenza vaccine (TIV) and/or 2009 monovalent pandemic vaccine effects on 2012–13 TIV effectiveness vs. A(H1N1)pdm09.**
(PDF)

Table S5 **Haemagglutinin antigenic site mutations in circulating A(H1N1)pdm09 viruses relative to the 2012–13 egg-adapted A/California/07/2009 X-179A high growth reassortant vaccine strain.**
(PDF)

Text S1 **Methods for haemagglutinin sequencing, phylogenetic and percent identity analysis.**
(PDF)
