# Supplementary material for: Low 2012–13 Influenza Vaccine Effectiveness Associated with Mutation in the Egg-Adapted H3N2 Vaccine Strain Not Antigenic Drift in Circulating Viruses
Source: PLoS One. 2014 Mar 25;9(3):e92153. doi: 10.1371/journal.pone.0092153 (PMC3965421; doi:10.1371/journal.pone.0092153)
Supplement: Figure S1 — Phylogenetic tree of influenza A/H3N2 viruses, sentinel system 2012–13. A maximum-likelihood phylogeny of the 152 sentinel viruses in the context of globally isolated 2012–2013 H3N2 viruses and recent vaccine components (n = 93) based on nucleotide alignment of the haemagglutinin HA1 domain is shown. Vaccine components and previously reported clades are labelled; sentinel viruses are coloured by province of origin. (PDF) [file pone.0092153.s001.pdf]

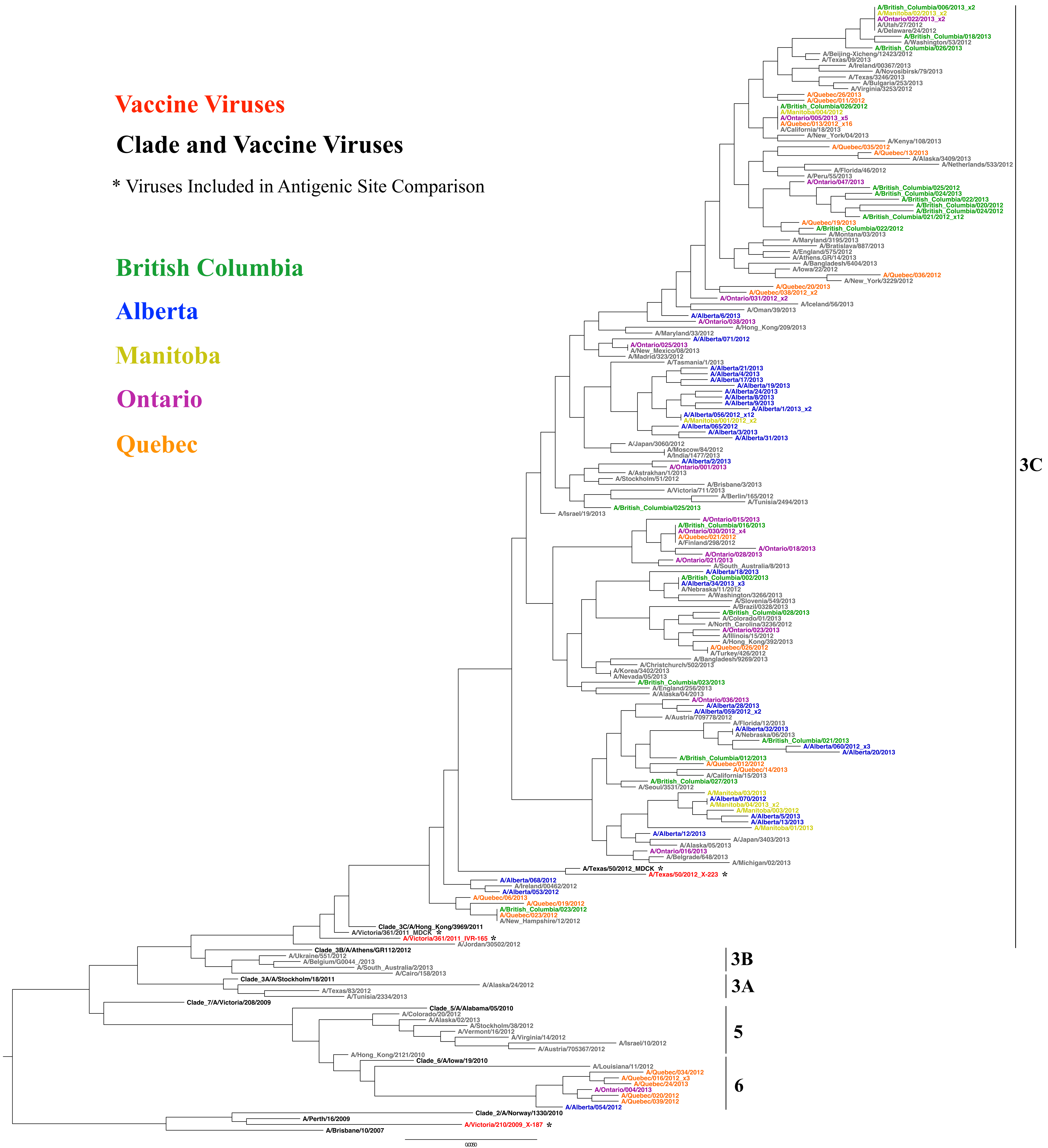

Vaccine Viruses
Clade and Vaccine Viruses
* Viruses Included in Antigenic Site Comparison
British Columbia
Alberta
Manitoba
Ontario
Quebec
A/British_Columbia/006/2013_x2
A/Manitoba/02/2013_x2
A/Ontario/022/2013_x2
A/Utah/27/2012
A/Delaware/24/2012
A/British_Columbia/018/2013
A/Washington/53/2012
A/British_Columbia/026/2013
A/Beijing-Xicheng/12423/2012
A/Texas/09/2013
A/Ireland/00367/2013
A/Novosibirsk/79/2013
A/Texas/3246/2013
A/Bulgaria/253/2013
A/Virginia/3253/2012
A/Quebec/26/2013
A/Quebec/011/2012
A/British_Columbia/026/2012
A/Manitoba/004/2012
A/Ontario/005/2013_x5
A/Quebec/013/2012_x16
A/California/18/2013
A/New_York/04/2013
A/Kenya/108/2013
A/Quebec/035/2012
A/Quebec/13/2013
A/Alaska/3409/2013
A/Netherlands/533/2012
A/Florida/46/2012
A/Peru/55/2013
A/Ontario/047/2013
A/British_Columbia/025/2012
A/British_Columbia/024/2013
A/British_Columbia/022/2013
A/British_Columbia/020/2012
A/British_Columbia/024/2012
A/British_Columbia/021/2012_x12
A/Quebec/19/2013
A/British_Columbia/022/2012
A/Montana/03/2013
A/Maryland/3195/2013
A/Bratislava/887/2013
A/England/575/2012
A/Athens.GR/14/2013
A/Bangladesh/6404/2013
A/Iowa/22/2012
A/Quebec/036/2012
A/New_York/3229/2012
A/Quebec/20/2013
A/Quebec/038/2012_x2
A/Ontario/031/2012_x2
A/Iceland/56/2013
A/Oman/39/2013
A/Alberta/6/2013
A/Ontario/038/2013
A/Hong_Kong/209/2013
A/Maryland/33/2012
A/Alberta/071/2012
A/Ontario/025/2013
A/New_Mexico/08/2013
A/Madrid/323/2012
A/Tasmania/1/2013
A/Alberta/21/2013
A/Alberta/4/2013
A/Alberta/17/2013
A/Alberta/19/2013
A/Alberta/24/2013
A/Alberta/8/2013
A/Alberta/9/2013
A/Alberta/1/2013_x2
A/Alberta/056/2012_x12
A/Manitoba/001/2012_x2
A/Alberta/065/2012
A/Alberta/3/2013
A/Alberta/31/2013
A/Japan/3060/2012
A/Moscow/84/2012
A/India/1477/2013
A/Alberta/2/2013
A/Ontario/001/2013
A/Astrakhan/1/2013
A/Stockholm/51/2012
A/Brisbane/3/2013
A/Victoria/711/2013
A/Berlin/165/2012
A/Tunisia/2494/2013
A/British_Columbia/025/2013
A/Israel/19/2013
A/Ontario/015/2013
A/British_Columbia/016/2013
A/Ontario/030/2012_x4
A/Quebec/021/2012
A/Finland/298/2012
A/Ontario/018/2013
A/Ontario/028/2013
A/Ontario/021/2013
A/South_Australia/8/2013
A/Alberta/18/2013
A/British_Columbia/002/2013
A/Alberta/34/2013_x3
A/Nebraska/11/2012
A/Washington/3266/2013
A/Slovenia/549/2013
A/Brazil/0328/2013
A/British_Columbia/028/2013
A/Colorado/01/2013
A/North_Carolina/3236/2012
A/Ontario/023/2013
A/Illinois/15/2012
A/Hong_Kong/392/2013
A/Quebec/026/2012
A/Turkey/426/2012
A/Bangladesh/9269/2013
A/Christchurch/502/2013
A/Korea/3402/2013
A/Nevada/05/2013
A/British_Columbia/023/2013
A/England/256/2013
A/Alaska/04/2013
A/Ontario/036/2013
A/Alberta/28/2013
A/Alberta/059/2012_x2
A/Austria/709778/2012
A/Florida/12/2013
A/Alberta/32/2013
A/Nebraska/06/2013
A/British_Columbia/021/2013
A/Alberta/060/2012_x3
A/Alberta/20/2013
A/British_Columbia/012/2013
A/Quebec/012/2012
A/Quebec/14/2013
A/California/15/2013
A/British_Columbia/027/2013
A/Seoul/3531/2012
A/Manitoba/03/2013
A/Alberta/070/2012
A/Manitoba/04/2013_x2
A/Manitoba/003/2012
A/Alberta/5/2013
A/Alberta/13/2013
A/Manitoba/01/2013
A/Alberta/12/2013
A/Japan/3403/2013
A/Alaska/05/2013
A/Ontario/016/2013
A/Belgrade/648/2013
A/Michigan/02/2013
A/Texas/50/2012_MDCK *
A/Texas/50/2012_X-223 *
A/Alberta/068/2012
A/Ireland/00462/2012
A/Alberta/053/2012
A/Quebec/06/2013
A/Quebec/019/2012
A/British_Columbia/023/2012
A/Quebec/023/2012
A/New_Hampshire/12/2012
Clade_3C/A/Hong_Kong/3969/2011
A/Victoria/361/2011_MDCK *
A/Victoria/361/2011_IVR-165 *
A/Jordan/30502/2012
3C
Clade_3B/A/Athens/GR112/2012
A/Ukraine/551/2012
A/Belgium/G0044_/2013
A/South_Australia/2/2013
A/Cairo/158/2013
3B
Clade_3A/A/Stockholm/18/2011
A/Alaska/24/2012
A/Texas/83/2012
A/Tunisia/2334/2013
3A
Clade_7/A/Victoria/208/2009
Clade_5/A/Alabama/05/2010
A/Colorado/20/2012
A/Alaska/02/2013
A/Stockholm/38/2012
A/Vermont/16/2012
A/Virginia/14/2012
A/Israel/10/2012
A/Austria/705367/2012
5
A/Hong_Kong/2121/2010
Clade_6/A/Iowa/19/2010
A/Louisiana/11/2012
A/Quebec/034/2012
A/Quebec/016/2012_x3
A/Quebec/24/2013
A/Ontario/004/2013
A/Quebec/020/2012
A/Quebec/039/2012
A/Alberta/054/2012
6
Clade_2/A/Norway/1330/2010
A/Perth/16/2009
A/Victoria/210/2009_X-187 *
A/Brisbane/10/2007
0.0050
