# Supplementary figures and images for: Low 2012–13 Influenza Vaccine Effectiveness Associated with Mutation in the Egg-Adapted H3N2 Vaccine Strain Not Antigenic Drift in Circulating Viruses
Source: PLoS One. 2014 Mar 25;9(3):e92153. doi: 10.1371/journal.pone.0092153 (PMC3965421; doi:10.1371/journal.pone.0092153)

Vaccine Virus

Clade Viruses

British Columbia

Alberta

Manitoba

Ontario

Quebec

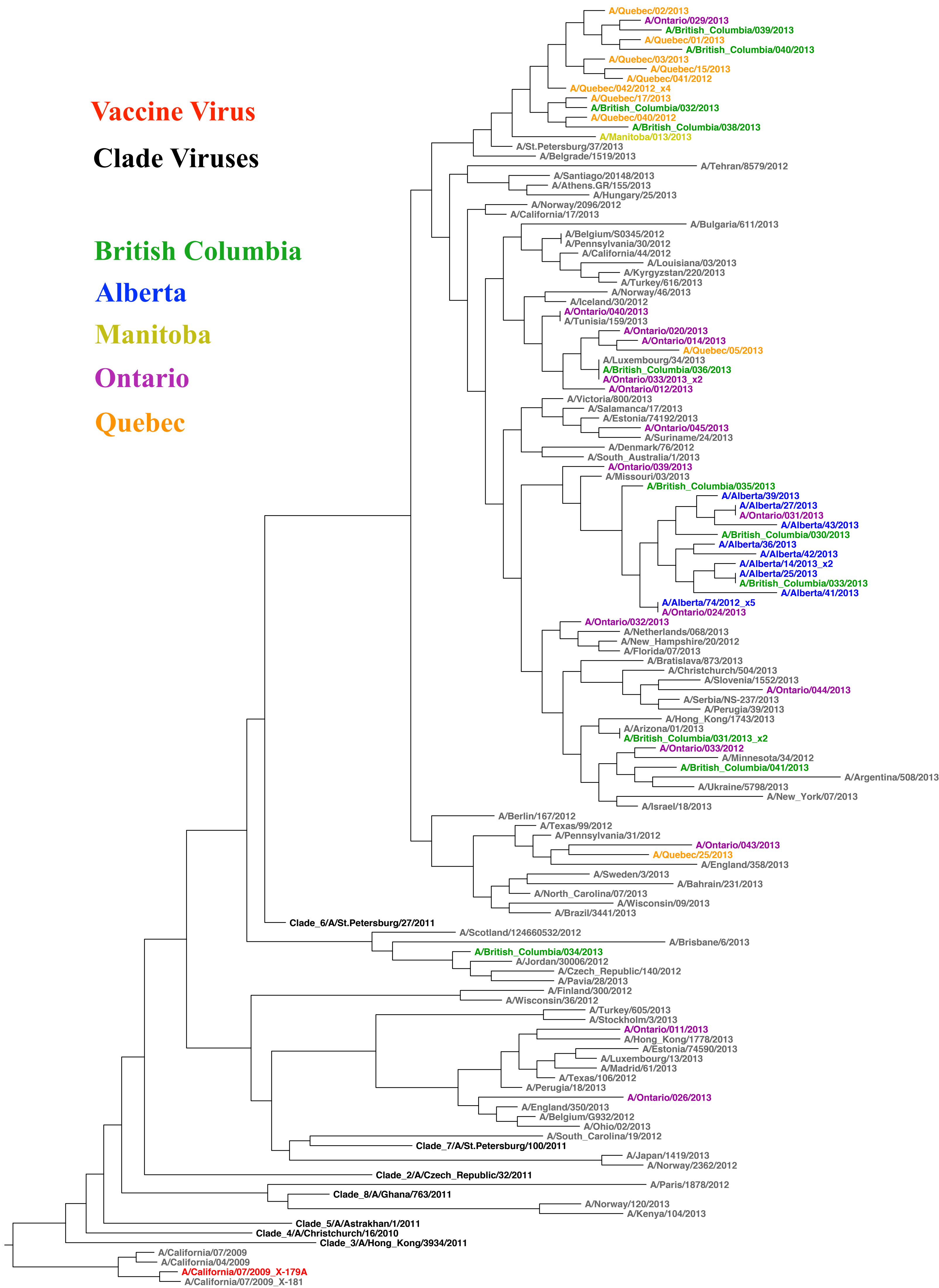

6

7

8

Supplement: Figure S2 — Phylogenetic tree of influenza A(H1N1)pdm09 viruses, sentinel system 2012–2013. A maximum-likelihood phylogeny of the 57 sentinel viruses in the context of globally isolated 2012–2013 A(H1N1)pdm09 viruses and recent vaccine components (n = 77) based on nucleotide alignment of the haemagglutinin HA1/HA2 domains is shown. Vaccine components and previously reported clades are labelled; sentinel viruses are coloured by province of origin. In September 2013, the European Centre for Disease Prevention and Control (ECDC) further divided clade 6 viruses into three genetic subgroups such that 1/55 (2%), 2/55 (4%) and 52/55 (95%) of the sentinel clade 6 A(H1N1)pdm09 viruses displayed belong to clade 6A, 6B and 6C. Subclade details are displayed in Table S5 [29]. (PDF) [file pone.0092153.s002.pdf]

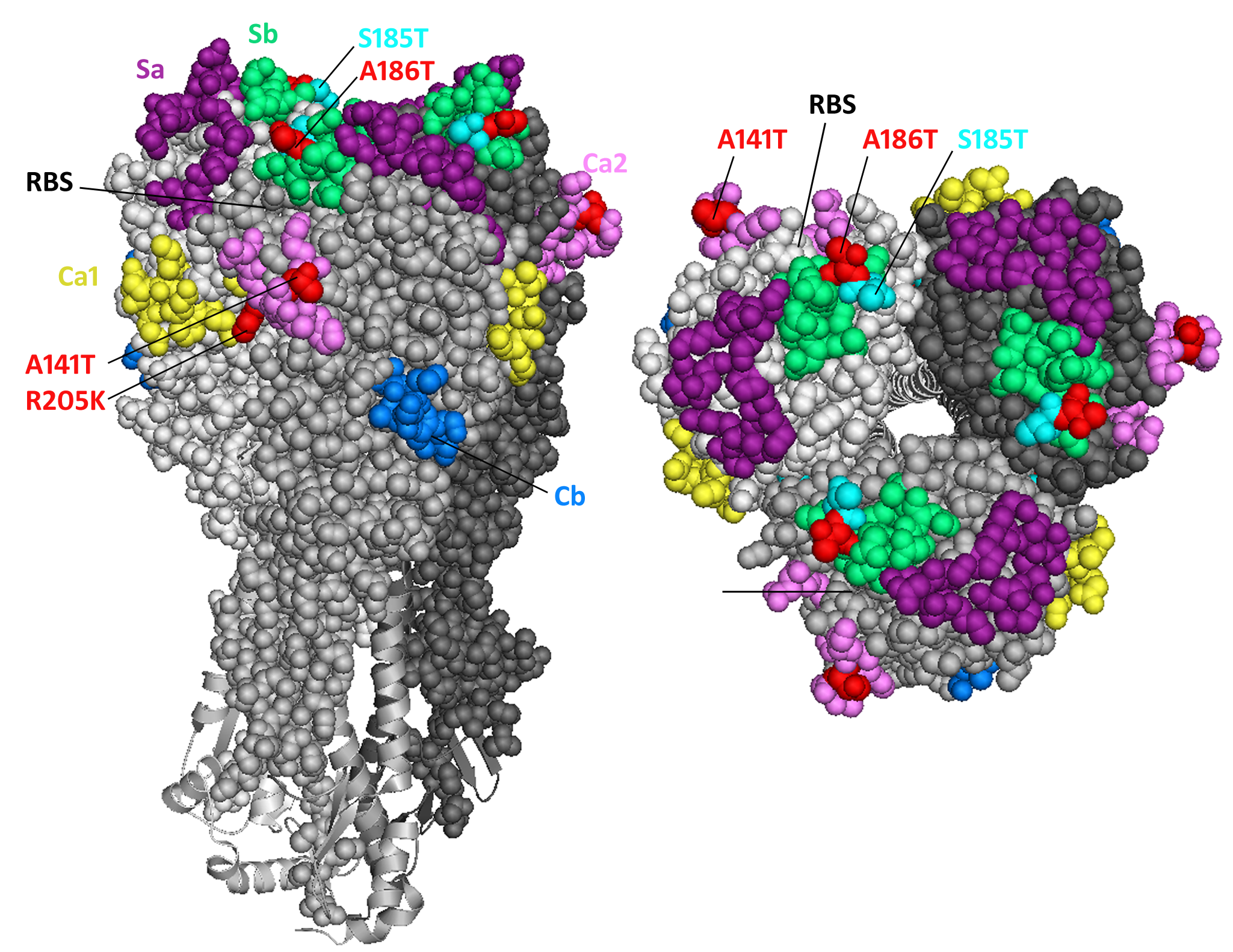

Supplement: Figure S3 — Three-dimensional model of antigenic-site mutations in circulating A(H1N1)pdm09 viruses relative to the 2012–13 egg-adapted A/California/04/2009 X-179A high growth reassortant vaccine strain. Three-dimensional structures of the trimeric haemagglutinin (HA) protein were constructed using the crystal structure of the A/California/04/2009(H1N1) HA (PDB, 3LZG) [30] Amino acid residues of the Sa, Sb, Ca1, Ca2 and Cb antigenic regions on the molecular surface (A: front view; B: top view) are colour-coded purple, green, yellow, pink and blue respectively and amino acid substitutions in circulating viruses relative to X-179A are labelled with coloured text representing their antigenic site positions. The three most prevalent mutations found in this study are coloured red (R205K site Ca1, A141T site Ca2, and A186T site Sb). Clade characteristic mutations S185T (representative for S185T/P) in antigenic site Sb and S203T (not visible in the figure) in antigenic site Ca are coloured cyan. RBS indicates approximate location of the receptor-binding site. (TIFF) [file pone.0092153.s003.tiff]
