# Supplementary material for: Low 2012–13 Influenza Vaccine Effectiveness Associated with Mutation in the Egg-Adapted H3N2 Vaccine Strain Not Antigenic Drift in Circulating Viruses
Source: PLoS One. 2014 Mar 25;9(3):e92153. doi: 10.1371/journal.pone.0092153 (PMC3965421; doi:10.1371/journal.pone.0092153)
Supplement: Table S1 — Influenza A H3 and H1 antigenic site maps. (PDF) [file pone.0092153.s004.pdf]

Table S1. Influenza A H3 and H1 antigenic site maps

a. H3 Antigenic site map (A-E: 131 amino acid residues) shown as amino acid (AA) number of the HA1 protein [1-3]

Antigenic Site (n=number of residues in total)	Residue Number(s)
A (n=19)	122, 124, 126, 130-133, 135, 137, 138, 140, 142-146, 150, 152, 168
B (n=22)	128, 129, 155-160, 163-165, 186-190, 192-194, 196-198
C (n=27)	44-48, 50, 51, 53, 54, 273, 275, 276, 278-280, 294, 297, 299, 300, 304, 305, 307-312
D (n=41)	96, 102, 103, 117, 121, 167, 170-177, 179, 182, 201, 203, 207-209, 212-219, 226-230, 238, 240, 242, 244, 246-248
E (n=22)	57, 59, 62, 63, 67, 75, 78, 80-83, 86-88, 91, 92, 94, 109, 260-262, 265

b. H1 Antigenic site map (Sa, Sb, Ca1, Ca2, and Cb: 50 amino acid residues) shown as AA number of the HA1 protein [2,4]

Antigenic Site (n=number of residues in total)	Residue Number(s)
Sa (n=13)	124, 125, 153-157, 159-164,
Sb (n=12)	184-195
Ca1 (n=11)	166-170, 203, 204, 205, 235-237
Ca2 (n=8)	137-142, 221, 222
Cb (n=6)	70-75

References S1:

1 Skowronski DM, Janjua NZ, De Serres G, Winter AL, Dickinson JA, et al. (2012) A sentinel platform to evaluate influenza vaccine effectiveness and new variant circulation, Canada 2010-11 season. Clin Infect Dis 55: 332–342.
2 Skowronski DM, Janjua NZ, Sabaiduc S, De Serres G, Winter A-L, et al. (2014) Influenza A/subtype and B/lineage effectiveness estimates for the 2011-12 trivalent vaccine: cross-season and cross-lineage protection with unchanged vaccine. J Infect Dis. Doi:10.1093/infdis/jiu048
3 Bush RM, Bender CA, Subbarao K, Cox NJ, Fitch WM (1999) Predicting the evolution of human influenza A. Science 286: 1921–1925.
4 Brownlee GG, Fodor E (2001) The predicted antigenicity of the haemagglutinin of the 1918 Spanish influenza pandemic suggests an avian origin. Phil Trans R Soc Lond 356: 1871–1876.
