# Supplementary material for: Low 2012–13 Influenza Vaccine Effectiveness Associated with Mutation in the Egg-Adapted H3N2 Vaccine Strain Not Antigenic Drift in Circulating Viruses
Source: PLoS One. 2014 Mar 25;9(3):e92153. doi: 10.1371/journal.pone.0092153 (PMC3965421; doi:10.1371/journal.pone.0092153)
Supplement: Table S2 — Proportion of participants who received 2012–13 TIV by age and influenza type, subtype and lineage. (PDF) [file pone.0092153.s005.pdf]

Table S2. Proportion of participants who received 2012-13 TIV[a] by age and influenza type, subtype and lineage

	Negative	Influenza	Influenza A	A/H3N2	A(H1N1)pdm09	Influenza B	B/Yamagata (vaccine)	B/Victoria (non-vaccine)
**Age (years)**	**n/N (%)**	**n/N (%)**	**n/N (%)**	**n/N (%)**	**n/N (%)**	**n/N (%)**	**n/N (%)**	**n/N (%)**
**1-8**	18/96 (19)	5/104 (5)	4/73 (5)	3/66 (5)	0/4 (0)	1/31 (3)	1/25 (4)	0/5 (0)
**9-19**	10/86 (12)	0/98 (0)	0/60 (0)	0/52 (0)	0/6 (0)	0/38 (0)	0/21 (0)	0/16 (0)
**20-49**	73/401 (18)	36/279 (13)	29/221 (13)	24/166 (14)	5/50 (10)	7/58 (12)	4/28 (14)	2/23 (9)
**50-64**	57/177 (32)	26/118 (22)	23/91 (25)	19/74 (26)	3/15 (20)	3/27 (11)	1/20 (5)	1/3 (33)
**65+**	66/89 (74)	28/53 (53)	22/40 (55)	20/36 (56)	2/4 (50)	6/13 (46)	3/4 (75)	2/7 (29)
**Total**	224/849 (26)	95/652 (15)	78/485 (16)	66/394 (17)	10/79 (13)	17/167 (10)	9/98 (9)	5/54 (9)

TIV=trivalent influenza vaccine. Note that where influenza A/subtype or B/lineage tallies do not sum to total influenza A or B detections, it is owing to missing subtype- or lineage-level information.

a. TIV receipt two weeks or more before onset of influenza-like symptoms per vaccine effectiveness analysis
