# Supplementary material for: Low 2012–13 Influenza Vaccine Effectiveness Associated with Mutation in the Egg-Adapted H3N2 Vaccine Strain Not Antigenic Drift in Circulating Viruses
Source: PLoS One. 2014 Mar 25;9(3):e92153. doi: 10.1371/journal.pone.0092153 (PMC3965421; doi:10.1371/journal.pone.0092153)
Supplement: Table S3 — Prior 2011–12 trivalent influenza vaccine (TIV) effects on current 2012–13 TIV effectiveness. (PDF) [file pone.0092153.s006.pdf]

Table S3. Prior 2011-12 trivalent influenza vaccine (TIV) effects on current 2012-13 TIV effectiveness

	Vaccine Effectiveness % (95% Confidence Interval (CI))						
		Influenza A and Subtype specific			Influenza B and Lineage specific		
	Any Influenza	Any Influenza A	A/H3N2	A/H1N1pdm09	Any Influenza B	B/Yamagata (vaccine)	B/Victoria (non-vaccine)
**N total;**	1403;	1246;	1161;	859;	941;	877;	834;
**n Cases; n Controls**	619; 784	462; 784	377; 784	75; 784	157; 784	93; 784	50; 784
Unadjusted [a]							
Unvaccinated 2011-12 and 2012-13	[Reference]	[Reference]	[Reference]	[Reference]	[Reference]	[Reference]	[Reference]
Current 2012-13 TIV only	67 (27 - 85)	72 (26 - 89)	64 (6 - 87)	NE	54 (-55 - 86)	48 (-125 - 88)	52 (-261 - 94)
Prior 2011-12 TIV only	15 (-20 - 40)	16 (-23 - 43)	-2 (-50 - 31)	NE	12 (-52 - 49)	-8 (-103 - 43)	24 (-97 - 71)
Both 2011-12 and 2012-13 TIV	51 (34 - 63)	42 (22 - 58)	39 (16 - 56)	NE	73 (51 - 86)	81 (53 - 93)	72 (22 - 90)
Adjusted [b]							
Unvaccinated in 2011-12 and 2012-13	[Reference]	[Reference]	[Reference]	[Reference]	[Reference]	[Reference]	[Reference]
Current 2012-13 TIV only	60 (9 - 82)	65 (8 - 87)	51 (-31 - 82)	NE	51 (-72 - 86)	33 (-202 - 85)	63 (-199 - 95)
Prior 2011-12 TIV only	8 (-32 - 36)	6 (-40 - 37)	-21 (-83 - 20)	NE	14 (-53 - 52)	-12 (-123 - 44)	35 (-81 - 76)
Both 2011-12 and 2012-13 TIV	46 (26 - 61)	39 (13 - 57)	34 (3 - 55)	NE	72 (44 - 86)	74 (30 - 90)	78 (26 - 94)

NE= Not estimable due to sparse data

a. Children < 2 years of age in 2012-13 were excluded from analysis as they may not have been vaccine-eligible during the fall 2011-12 immunization campaign on the basis of age <6 months.

b. Adjusted for: age (2-8, 9-19, 20-49, 50-64, ≥65 years), comorbidity, province, interval, week
