# Supplementary material for: Low 2012–13 Influenza Vaccine Effectiveness Associated with Mutation in the Egg-Adapted H3N2 Vaccine Strain Not Antigenic Drift in Circulating Viruses
Source: PLoS One. 2014 Mar 25;9(3):e92153. doi: 10.1371/journal.pone.0092153 (PMC3965421; doi:10.1371/journal.pone.0092153)
Supplement: Table S4 — Prior 2011–12 trivalent influenza vaccine (TIV) and/or 2009 monovalent pandemic vaccine effects on 2012–13 TIV effectiveness vs. A(H1N1)pdm09. (PDF) [file pone.0092153.s007.pdf]

Table S4. Prior 2011-12 trivalent influenza vaccine (TIV) and/or 2009 monovalent pandemic vaccine[a] effects[b] on 2012-13 TIV effectiveness vs. A(H1N1)pdm09

	Vaccine effectiveness % (95% CI)	
**N total = 758; n Cases =67; n Controls=691**	**Crude (unadjusted)**	**Adjusted [c]**
Unvaccinated each specified year (none of 3 vaccines)	[Reference]	[Reference]
2009 monovalent A(H1N1)pdm09 vaccine only	55 (2 - 79)	51 (-8 - 78)
(2011-12 OR 2012-13 TIV) but not both, AND NOT 2009 monovalent A(H1N1)pdm09 vaccine	NE	NE
(2011-12 OR 2012-13 TIV) but not both, AND 2009 monovalent A(H1N1)pdm09 vaccine	79 (11 - 95)	79 (9 - 95)
Both 2011-12 AND 2012-13 TIV AND NOT 2009 monovalent A(H1N1)pdm09 vaccine	72 (-116 - 96)	76 (-83 - 97)
Both 2011-12 AND 2012-13 TIV AND 2009 monovalent A(H1N1)pdm09 vaccine (all 3 vaccines)	67 (26 - 86)	63 (12 - 85)

a. AS03-adjuvanted A(H1N1)pdm09 vaccine was predominantly distributed (>95% of doses) during the 2009 monovalent pandemic influenza immunisation campaign in Canada.
b. Children < 4 years of age in 2012-13 were excluded from analysis as they may not have been vaccine-eligible during the fall 2009 immunization campaign on the basis of age <6 months.
c. Adjusted for age (4-8, 9-19, 20-49, ≥50 years), comorbidity, interval, week
