# Supplementary material for: Low 2012–13 Influenza Vaccine Effectiveness Associated with Mutation in the Egg-Adapted H3N2 Vaccine Strain Not Antigenic Drift in Circulating Viruses
Source: PLoS One. 2014 Mar 25;9(3):e92153. doi: 10.1371/journal.pone.0092153 (PMC3965421; doi:10.1371/journal.pone.0092153)
Supplement: Table S5 — Haemagglutinin antigenic site mutations in circulating A(H1N1)pdm09 viruses relative to the 2012–13 egg-adapted A/California/07/2009 X-179A high growth reassortant vaccine strain. (PDF) [file pone.0092153.s008.pdf]

Table S5. Haemagglutinin (HA) antigenic site differences in circulating A(H1N1)pdm09 viruses relative to the 2012-13 egg-adapted A/California/07/2009 X-179A high growth reassortant vaccine strain

Antigenic Site		Cb	Ca2		Sa	Ca1	Sb		Ca1			# of AA	
HA1 Position		74	138	141	163	170	185	186	203	205	Clade	differences[a]	% AA identity[a]
A/California/07/2009		S	H	A	K	G	S	A	S	R	-	0	100%
**A/California/07/2009 X-179A[b]**		S	H	A	K	G	S	A	S	R	-	-	-
**British Columbia**	**N**												
A/British Columbia/30/2013	2						**T**		**T**	**K**	6C	3	94.0%
A/British Columbia/31/2013	3						**T**		**T**		6C	2	96.0%
A/British Columbia/32/2013	4			**T**			**T**		**T**		6C	3	94.0%
A/British Columbia/33/2013	1						**P**		**T**	**K**	6C	3	94.0%
A/British Columbia/34/2013	1		**R**				**T**		**T**		6A	3	94.0%
A/British Columbia/36/2013	1						**T**	**T**	**T**		6C	3	94.0%
**Alberta**	**N**												
A/Alberta/74/2012	11						**T**		**T**	**K**	6C	3	94.0%
A/Alberta/14/2013	3						**P**		**T**	**K**	6C	3	94.0%
**Manitoba**	**N**												
A/Manitoba/13/2013	1			**T**			**T**		**T**		6C	3	94.0%
**Quebec**	**N**												
A/Quebec/040/2012	11			**T**			**T**		**T**		6C	3	94.0%
A/Quebec/05/2013	1						**T**	**T**	**T**		6C	3	94.0%
A/Quebec/25/2013	1				**Q**		**T**		**T**		6B	3	94.0%
**Ontario**	**N**												
A/Ontario/033/2012	4						**T**		**T**		6C	2	96.0%
A/Ontario/012/2013	6						**T**	**T**	**T**		6C	3	94.0%
A/Ontario/024/2013	3						**T**		**T**	**K**	6C	3	94.0%
A/Ontario/029/2013	1	**I**		**T**			**T**		**T**		6C	4	92.0%
A/Ontario/043/2013	1				**Q**		**T**		**T**	**K**	6B	3	94.0%
A/Ontario/011/2013	1				**I**	**R**	**T**		**T**		7	4	92.0%
A/Ontario/026/2013	1				**I**		**T**		**T**		7	3	94.0%

N=number of sentinel viruses with that sequence. Bold font signifies amino acid (AA) substitution compared with A/California/07/2009 X-179A. Clade designation, number of antigenic site differences and percent antigenic site pairwise identity are also displayed. Antigenic regions comprise 50 AA residues but only the 9/50 positions showing differences between circulating A(H1N1)pdm09 and X-179A are displayed.

a. In September 2013, the European Centre for Disease Prevention and Control (ECDC) further divided clade 6 viruses into three genetic subgroups such that 1/55 (2%), 2/55 (4%) and 52/55 (95%) of sentinel clade 6 A(H1N1)pdm09 viruses during 2012-13 belong to clade 6A, 6B and 6C, respectively [29].
b. A/California/07/2009 X-179A (hereafter "X-179A") is one of the egg-adapted high growth reassortant vaccine strains substituted by manufacturers for the A/California/07/2009 strain recommended by the World Health Organization as 2012-13 vaccine component. X-181 is an alternate available vaccine strain. Both X-179A and X-181 are identical in their antigenic sites to each other and to A/California/07/2009, with a single non-antigenic site substitution in X-181 (N129D). The number of antigenic site AA differences and percent antigenic site identity are presented here relative to X-179A. Percent identity was derived as per **Text S1**.
