# Supplementary material for: Low 2012–13 Influenza Vaccine Effectiveness Associated with Mutation in the Egg-Adapted H3N2 Vaccine Strain Not Antigenic Drift in Circulating Viruses
Source: PLoS One. 2014 Mar 25;9(3):e92153. doi: 10.1371/journal.pone.0092153 (PMC3965421; doi:10.1371/journal.pone.0092153)
Supplement: Text S1 — Methods for haemagglutinin sequencing, phylogenetic and percent identity analysis. (PDF) [file pone.0092153.s009.pdf]

S1. Methods for haemagglutinin sequencing, phylogenetic and percent identity analysis

Sequencing of the HA1 gene of a subset (n=152) of available influenza A/H3N2 viruses, and the HA1/HA2 of A(H1N1)pdm09 viruses (n=57) spanning the season was undertaken for each province to identify potentially relevant amino acid (AA) substitutions. Virus was sequenced from culture isolates (Ontario) or original patient specimens (all provinces including Ontario in the event virus could not be cultivated). Alignments of translated protein sequences were generated in Geneious Pro v4.8.5 [1] using the MUSCLE multiple sequence alignment algorithm. Percent identity was calculated as [1-(number of AA substitutions in antigenic sites)/(total antigenic site AA residues)]X100%. Phylogenetic trees of nucleotide sequences were generated using the approximate likelihood method FastTree v2.1 [2], run using the Jones-Taylor-Thornton (JTT) model of AA evolution and a combination of nearest- neighbour interchanges (NNIs) and subtree-prune-regraft moves (SPRs) for branch swapping. The Shimodara-Hasegawa test on alternate topologies was used as a fast approximate likelihood ratio test to quantify branch support, with replicates set to 1,000. Trees were visualized using FigTree [3] and rooted using the oldest sequence.

A/H3N2

For A/H3, pairwise identities were calculated from the translated protein sequences aligned to the vaccine reference strain (A/Victoria/361/2011 IVR-165), displayed also in relation to the Madin Darby Canine Kidney (MDCK) cell-passaged A/Victoria/361/2011 reference strain recommended by the World Health Organization (WHO) for 2012-13 and the WHO-recommended strain for 2013-14 both MDCK cell- (A/Texas/50/2012) and egg-passaged (A/Texas/50/2012 X-223) as well as the vaccine strain used by manufacturers in 2011-12 (A/Victoria/210/2009 X-187). The resulting alignment of 157 sequences was manually inspected and trimmed to remove the signal peptide (residues 1-16) and the HA2 domain (residues 346-566). Since the H3 antigenic sites A-E are of greatest interest in correlating with vaccine effectiveness, pairwise identities were only calculated and reported for the 131 AA residues that make up the antigenic sites shown in the **Table S1** antigenic map (as previously published [4,5] and derived from the work of Bush and colleagues) [6].

For the phylogenetic tree, the 152 sentinel H3N2 sequences were aligned against: i) 7 sequences reported in the June 2012 CNRL Technical Document [7] specifying H3N2 clade nomenclature; ii) the A/Hong Kong/2121/2010 sequence previously dominant during the 2010-11 sentinel season [4]; iii) sequences of the 2007-2013 H3N2 vaccine components A/Brisbane/10/2007, A/Perth/16/2009, A/Victoria/361/2011 and A/Texas/50/2012; and iv) 86 global sequences collected from November 1, 2012 to August 14, 2013 obtained from the Global Initiative on Sharing All Influenza Data (GISAID) EpiFlu Database [8]. Sequences were trimmed to remove the signal peptide and HA2 domain and a phylogenetic tree was generated in FastTree [2]. All sequences in the alignment were complete over the full length of the HA1 domain.

A(H1N1)pdm09

For A(H1N1)pdm09, pairwise identities for virus were calculated from the translated protein sequences of HA1/HA2 aligned to the vaccine reference strain (A/California/07/2009 X-179A) in Geneious Pro [1]. After removal of the signal peptide (residues 1-17), the percent pairwise identities were calculated as described above in relation to established HA1 Sa, Sb, Ca1, Ca2, and Cb, antigenic sites (50AA) displayed in **Table S1** [5,9]. For the phylogenetic tree, the 57 sentinel A(H1N1)pdm09 sequences were aligned against: i) 8 sequences reported in the March 2012 CNRL Technical Document [10] specifying A(H1N1)pdm09 clade nomenclature; ii) 3 A/California/07/2009 vaccine strains; and iii) 74 global sequences collected from November 1, 2012 to August 14, 2013 obtained from GISAID [8]. The signal peptide was removed from the alignment and a phylogenetic tree was generated in FastTree [2].

References S1

1 Drummond AJ, Ashton B, Cheung M, Heled J, Kearse M, et al. (2009) Geneious v4.8.5. Available: http://www.geneious.com/
2 Price M, Dehal P, Arkin A (2010) FastTree 2 - approximately maximum-likelihood trees for large alignments. PLoS ONE 5: e9490. doi: 10.1371/journal.pone.0009490.
3 Rambaut A. FigTree v1.4.0, a graphical viewer of phylogenetic trees. Edinburgh: University of Edinburgh. Available: http://tree.bio.ed.ac.uk/software/figtree/

4 Skowronski DM, Janjua NZ, De Serres G, Winter AL, Dickinson JA, et al. (2012) A sentinel platform to evaluate influenza vaccine effectiveness and new variant circulation, Canada 2010-11 season. Clin Infect Dis 55: 332–342.

5 Skowronski DM, Janjua NZ, Sabaiduc S, De Serres G, Winter A-L, et al. (2014) Influenza A/subtype and B/lineage effectiveness estimates for the 2011-12 trivalent vaccine: cross-season and cross-lineage protection with unchanged vaccine. J Infect Dis Doi:10.1093/infdis/jiu048

6 Bush RM, Bender CA, Subbarao K, Cox NJ, Fitch WM (1999) Predicting the evolution of human influenza A. Science 286: 1921–1925.

7 Influenza Virus Characterization, Summary Europe, June 2012. Technical Document. Community Network of Reference Laboratories for Human Influenza in Europe. Available: http://www.ecdc.europa.eu/en/publications/Publications/Influenza-visus-characterisation-June-2012.pdf Accessed 7 December 2013.

8 Available: http://platform.gisaid.org/

9 Brownlee GG, Fodor E (2001) The predicted antigenicity of the haemagglutinin of the 1918 Spanish influenza pandemic suggests an avian origin. Phil Trans R Soc Lond 356: 1871–1876.

10 Influenza Virus Characterization, Summary Europe, March 2012. Technical Document. Community Network of Reference Laboratories for Human Influenza in Europe. Available: http://ecdc.europa.eu/en/publications/Publications/1204-TED-CNRL-report.pdf. Accessed 7 December 2013.
